# Cleavable Silyl Ether Monomers with Elevated Thermomechanical Properties for Bone Regeneration

**DOI:** 10.1021/acsabm.5c01174

**Published:** 2025-11-07

**Authors:** Tina Gurmann, Judith Krauß, Theresa Ammann, Thomas Koch, Martin Frauenlob, Robert Liska, Stefan Baudis

**Affiliations:** † Christian Doppler Laboratory for Advanced Polymers for Biomaterials and 3D Printing, 1060 Vienna, Austria; ‡ Institute of Applied Synthetic Chemistry, 27259 Technische Universität Wien, 1060 Vienna, Austria; § Austrian Cluster for Tissue Regeneration, 1200 Vienna, Austria; ∥ Institute of Materials Science and Technology, 27259 Technische Universität Wien, 1060 Vienna, Austria

**Keywords:** biomaterials, bone tissue engineering, photopolymerization, additive manufacturing, degradability

## Abstract

Over the last years, stereolithography has developed to be one of the most promising fabrication techniques in tissue engineering. Posing the possibility of fabricating patient-specific, porous implants, it became especially attractive for scaffold fabrication for the treatment of critical sized bone defects. State-of-the-art photopolymer systems mostly consist of potentially cytotoxic compounds, such as (meth)­acrylates, that furthermore show insufficient degradation and lead to acidic degradation products that could induce adverse tissue reactions. Herein, we introduced trifunctional monomers comprising cleavable silyl ether groups for thiol–ene photopolymerization to enlarge the material platform for printed bone grafts. Polymer networks comprising a high number of silyl ether moieties typically tend to be mechanically weak and exhibit low *T*_g_ values, especially when combined with thioether bonds, which are a direct result of polymerization via thiol–ene click reaction. To push thermomechanical properties to a level where they are sufficient for bone grafting (*T*_g_ > 37 °C), we introduced rigid bridged alicyclic structures in the form of norbornane-derived motifs into the silyl ether monomers, resulting in a norbornene-containing double bond monomer and a norbornane-derived thiol monomer. Together with noncleavable comonomers, we were able to demonstrate a substantial increase in *T*_g_ up to 62 °C, which is well above the values reported until now for similar thiol–ene networks. Furthermore, in this study, we demonstrated high photoreactivity for some of the monomers and also successfully performed proof-of-concept printing using a DLP setup. Besides excellent thermomechanical behavior, the mechanical strength of the silyl ether-based polymer network was shown to be outstanding. Cleavability of the silyl ethers was displayed with a quasi-linear degradation rate of 6.5% per month with moderate swelling. Additionally, the degradation product of the silyl ether-based network was isolated and shown to exhibit no relevant cytotoxicity to mouse fibroblast cells.

## Graphical abstract

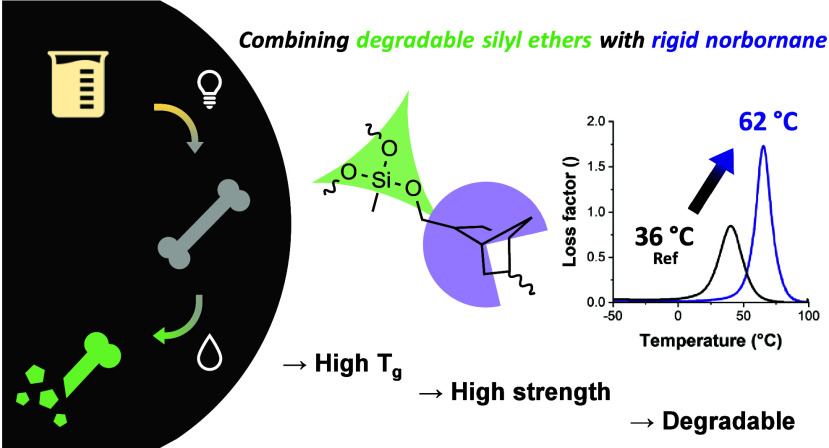

## 1 Introduction

Within the last 30 years, the number of new bone fractures per year has substantially increased. More specifically, a total of 178 million bone fractures was counted for the year 2019, which resulted in an approximate increase of one-third compared to the year 1990. As a consequence, major socioeconomic burdens, such as decreased productivity, poorer quality of life, and high costs for healthcare, have to be tackled nowadays.^1^ Due to the high need and constant growth of the market, research toward improved tissue regeneration already started in the early 1970s with the first attempts to generate cartilage tissue.^2^ Today, modern tissue engineering and regenerative medicine relies on the three pillars of tissue engineering: cells, signals, and scaffolds. Combination of these three pillars is one of the pursued approaches to achieve optimal results for tissue regeneration.^3,4^ Nevertheless, autologous bone grafts are still considered the “gold standard” when it comes to the treatment of critical-sized bone defects, even though bone graft harvesting is associated with a wide range of complications including infections, nerve and vascular injuries, chronic donor site pain, scarring, etc.^4–6^ Hence, finding a suitable material as an alternative would reduce the mentioned socioeconomic burdens. The variety of possible materials is as diverse as the range of materials themselves: from ceramics to metals or polymers of natural or synthetic origin.^4,7–10^ One material class which is especially attractive for scaffold engineering is polymers, as they are highly versatile and can be tuned toward desired properties, e.g., improved cell adhesion,^11^ antimicrobial properties,^12^ or enhanced mechanical properties.^13^ Commonly used synthetic thermoplastic polymers include poly­(lactic acid) (PLA) and poly­(ε-caprolactone) (PCL).^4,7–10,14^ Although both of them exhibit favorable properties such as good availability, easy processability, and sufficient biocompatibility, they also exhibit certain drawbacks. One major obstacle with PLA and PCL is their degradation behavior. Upon hydrolytic degradation, the polymer backbones are cleaved, which results in degradation products with acidic moieties for both PLA and PCL. This leads to a local drop of the pH which has two major consequences. On the one hand, polymer degradation is accelerated due to autocatalytic effects, leading to unfavorable bulk erosion of the material. Bulk erosion typically causes deterioration of the mechanical properties and premature implant failure. Second, an acidic environment may trigger an adverse bone response and, therefore, excessive demineralization of surrounding tissue.^8,9,15^

Furthermore, PLA and PCL are not suitable for a fabrication method that arose over the course of the past decade, stereolithography (SLA)-based additive manufacturing (AM), which holds several advantages compared to other fabrication techniques (e.g., higher resolution, manufacturing of complex geometries).^10^ For SLA, (photo)­polymerizable groups are required to form covalent bonds upon irradiation. Commercially available resins mostly include (meth)­acrylate-based monomers that exhibit very good reactivity but result in rather weak and brittle materials. In addition to the toxicity of unreacted monomers, poly­((meth)­acrylic acids) are formed during degradation, which are known to promote inflammatory responses.^16–18^ In the past, several alternatives for cleavable monomers with enhanced cytocompatibility have been explored, including vinyl esters,^16,19^ (cyclic) acetals,^20–22^ boronic esters,^17^ and silyl ethers.^23–26^ Extensive studies have been performed regarding the tunability of silyl ether degradability by adjusting the bulkiness and hydrophobicity of side groups at the silicon central atom.^27^ Parrott et al. demonstrated the influence of steric hindrance on the degradation rate by introduction of methyl, ethyl, isopropyl, and *tert*-butyl groups to the silicon atom of a bifunctional acrylate-based cross-linker. The resulting materials differed in their degradation rates by orders of magnitude with the methyl derivative having the fastest degradation rate.^25^ Bunton et al. showed the same effect with trifunctional acrylate and thiol monomers, but here they used methyl and phenyl groups.^24^

Since the introduction of silyl ether linkages as dynamic motifs in vitrimers by Nishimura and co-workers, this employment of silyl ethers has been thoroughly discussed in literature.^28–32^ Besides demonstrating their applicability as a vitrimeric motif, Nishimura et al. also showed the importance of neighboring groups (i.e., γ-amino groups) for appropriate exchange kinetics.^28^ Another use of silyl ethers was proposed by Johnson and Johnson, who introduced cleavable silyl ether motifs into a high-density poly­(ethylene) (HDPE) backbone. This was done by a catalytic one-pot ring-opening metathesis polymerization of a cyclic silyl ether and cyclooctene, followed by hydrogenation of the copolymer backbone, resulting in an HDPE-like polymer. The labile bond allowed chemical de- and reconstruction of the linear polymer and therefore presented a promising approach for future “closed-loop” recycling of PE.^33^ In a recent publication, Teasdale’s group employed silyl ether-based thiol monomers for inkjet printing formulations for biomedical applications. Here, the flexible Si–O bond was utilized to lower the formulation viscosity and enhance the printing quality. They demonstrated good printability of the silyl ether-containing systems and showed promising biocompatibility.^34^ Although silyl ethers have shown sufficient and tunable degradation properties, previously, they exhibited rather poor (thermo)­mechanical properties, especially in combination with thiol–ene click chemistry, for application in bone tissue engineering. Thermosets or elastomers that comprise silyl ether motifs are known to be rather mechanically weak and to have a low glass transition temperature, *T*_g_. Upon addition of another mechanically rather weak bond, such as the thioether bond, which is introduced during thiol–ene photopolymerization, the mechanical properties of the final networks suffer even more.^23,24,34^ For instance, Ware et al. introduced silyl ether-based monomers to thiol–ene click chemistry and demonstrated great tunability of network properties by variation of substituents on the silicon central atom.^23^ Although it was possible to tune the *T*_g_ up to 40 °C, such materials would still be insufficient as bone scaffolds, as the storage modulus *G*′ was already declining at temperatures lower than 37 °C.

To increase stiffness, rigidity, and thermomechanical behavior, rigid structures could be incorporated into the monomers’ chemical structure. One promising concept for linear silyl ether-based polymers was presented by the group of Du, where they showed an increase in *T*_g_ by incorporation of bicyclic structures in the form of isosorbide groups into the polymer backbone.^35^

Inspired by this concept of limiting freely rotatable C–C bonds, norbornene side groups were introduced to the structure of silyl ether monomers. Due to the methylene bridge contained in the basic norbornane structure, the six-membered ring is trapped in its boat conformation and should therefore contribute rigidity to the network.^36,37^ To examine the influence of norbornane motifs on the polymer network behavior, we synthesized and polymerized two silyl ether-based monomers, one ene and one thiol compound. For thorough investigation, the silyl ether monomers were also polymerized with comonomers that did not contain silyl ether or norbornane structures. The resulting formulations and materials were tested regarding their photoreactivity, as this is a crucial parameter for 3D-printing, and their (thermo)­mechanical properties. Furthermore, the hydrolytic degradation behavior was studied as well as the cytotoxicity of degradation products of the most promising polymer network in terms of (thermo)­mechanical properties. In the end, the printability of this formulation was demonstrated.

## 2 Experimental Section

### 2.1 Reagents and Materials

(5-Norbornene-2-yl)­methanol (distilled), thioacetic acid (TCI, >95%), trichloro­(methyl)­silane (TCI, >98%), 1,2,4-trivinylcyclohexane (Sigma-Aldrich, 98%), triethylamine (distilled, Sigma-Aldrich, >99%), and 2,2′-azobis­(2-methylpropionitrile) (AIBN, recrystallized from MeOH at rt, Sigma-Aldrich, 98%) were purchased from the respective companies and used as received unless stated otherwise. Dry tetrahydrofuran (THF, Fisher Chemical, 99.8%) and dry diethyl ether (Honeywell) were dried using a PureSolv system (Inert, Amesbury, MA). Dichloromethane (DCM, Fisher Chemical), methanol (Sigma-Aldrich, p.a.), conc. hydrochloric acid (HCl, Sigma-Aldrich, 37%), petroleum ether (Fisher Scientific), and ethyl acetate (Sigma-Aldrich, >99.5%) were purchased from the respective companies. The photoinitiator bis­(4-methoxybenzoyl)­diethylgermanium (Ivocerin) was kindly provided by Ivoclar Vivadent AG and used as received. Unless further described, all reagents used in assessing cytotoxicity were purchased from Thermofisher Scientific and used as received. The mouse fibroblast cell line NCTC 929 was acquired from LGC Limited, a supplier of the ATCC biobank.

### 2.2 Synthesis of *C*-Methyl­(hydroxymethyl)-2-norbornanecarbothioate (Mixture of Isomers, NMTA)

The described synthesis pathway was adapted from a method described by Reinelt et al.^38,39^ The title substance was synthesized starting from distilled (5-norbornene-2-yl)­methanol (30 g, 1 equiv, 241.8 mmol) and thioacetic acid (22.1 g, 1.2 equiv, 289.9 mmol). Both reagents were diluted in 250 mL of dry THF in a 500 mL three-necked flask equipped with a magnetic stirring bar and reflux condenser. Subsequently, AIBN, recrystallized from methanol (2.0 g, 0.05 equiv, 12.1 mmol), was added. The solution was purged with argon for 30 min and then heated to reflux for 24 h. For workup, the cooled reaction mixture was diluted with 200 mL of 1 M sodium carbonate solution and then extracted three times with DCM (200/100/100 mL). The organic phase was then extracted with 50 mL of 1 M sodium hydroxide solution and 50 mL of brine. Afterward, residual moisture was removed from the organic phase with sodium sulfate (anhydrous). After filtration and washing, solvents were removed in vacuo to obtain the dark brown to rust-colored liquid product in quantitative yield (48.5 g). ^13^C NMR data can be found in the SI.

^1^H NMR (400 MHz, CD_2_Cl_2_) δ 3.59–3.08 (m, 2H, -O-CH_2_-), 2.37–2.22 (m, 1H, aliphatic H), 2.17 (d, *J* = 2.8 Hz, 3H, -CH_3_), 2.11–1.88 (m, 2H, aliphatic H), 1.82–1.65 (m, 1H, aliphatic H), 1.62–0.88 (m, 5H, aliphatic Hs), 0.74 (ddd, *J* = 12.8, 5.2, 2.5 Hz, 1H, aliphatic H).

### 2.3 Synthesis of (Mercapto-2-norbornanyl)­methanol (Mixture of Isomers, NMT)

To obtain the monomer precursor NMT, NMTA was hydrolyzed as described in the literature.^38,39^ For this, NMTA (46.1 g, 1 equiv, 230.0 mmol) was transferred to a 1000 mL three-necked flask and diluted with 600 mL of methanol. Conc. HCl (43 mL) was added subsequently. The reaction solution was purged with argon for 30 min to prevent the oxidation of the resulting free thiols. After this, the solution was refluxed for 24 h. 250 mL of deionized water was added to the cooled reaction mixture, which was then extracted three times with DCM (450/250/250 mL). The combined organic phases were then washed twice with 250 mL of saturated sodium bicarbonate solution and then once with 250 mL of brine. The organic phase was further dried with sodium sulfate (anhydrous). After the solvent was removed in vacuo, the product was obtained as a dark yellow oil (32.0 g, 88% yield). ^13^C NMR can be found in the SI.

^1^H NMR (400 MHz, CD_2_Cl_2_) δ 3.75–3.26 (m, 2H, -O-CH_2_-), 3.22–2.67 (m, 1H, -CH-SH)), 2.37–1.95 (m, 3H, aliphatic Hs, -SH), 1.87–1.53 (m, 3H, aliphatic Hs), 1.51 (s, 1H, -OH), 1.47–0.93 (m, 3H, aliphatic Hs), 0.62 (dddd, *J* = 27.0, 12.3, 5.3, 2.5 Hz, 1H, aliphatic H).

### 2.4 Synthesis of 6-((Bis­((6-mercapto-2-norbornanyl)­methoxy)­(methyl)­siloxy)­methyl)-2-norbornanethiol (Mixture of Isomers, TSE)

The synthesis of TSE was adapted from the literature^23^ and started by charging a predried 1000 mL three-necked flask, equipped with a mechanical stirrer, with 480 mL of dry diethyl ether and freshly distilled triethylamine (19.3 g, 3 equiv, 191.1 mmol). The flask was cooled by using an ice bath. Trichloro­(methyl)­silane (9.5 g, 1 equiv, 63.7 mmol) was slowly added via syringe under argon counterflow and vigorous stirring, followed by NMT (30.2 g, 3 equiv, 191.1 mmol) which was diluted under inert conditions with 50 mL of dry ether beforehand. After the complete addition of all reagents, the contents of the flask were slowly warmed to rt. Stirring was continued under argon for 24 h. Following this, the formed precipitate was filtered off, and the solvent was removed in vacuo. For purification, the crude product was flashed over neutral aluminum oxide using PE/EE (20:1). The product was obtained as a yellow oil (16.8 g, 51% yield, mixture of isomers). Purity was shown via UHPLC-MS (see SI).

^1^H NMR (400 MHz, CD_2_Cl_2_) δ 3.92–3.37 (m, 6H, -O-CH_2_-), 3.30–2.63 (m, 3H, -CHR_2_-SH), 2.39–0.28 (m, 27H, aliphatic Hs), 1.52 (m, 3H, -SH), 0.09 (dh, *J* = 11.3, 3.6 Hz, 3H, R_3_Si–CH_3_).

^13^C NMR (101 MHz, CD_2_Cl_2_) δ 64.23 (-O-CH_2_-), 63.86 (-O-CH_2_-), 48.87 (-CH-CHR-CH=), 47.71 (-CH-CHR-CH=), 43.38 (aliphatic C), 42.68 (aliphatic C), 41.22 (aliphatic C), 40.87 (aliphatic C), 40.28 (aliphatic C), 39.42 (aliphatic C), 37.90 (aliphatic C), 36.77 (aliphatic C), 36.61 (aliphatic C), 35.97 (aliphatic C), 34.35 (aliphatic C), 33.02 (aliphatic C), 32.00 (aliphatic C). ^29^Si NMR (80 MHz, CD_2_Cl_2_) δ −41.94. *R*_f_ (AlOx neutral, PE/EE = 20:1): 0.84.

### 2.5 Synthesis of Methyltris­((5-norbornen-2-yl)­methoxy)­silane (Mixture of Isomers, NSE)

The norbornene monomer was synthesized as described for TSE using distilled (5-norbornene-2-yl)­methanol (9.0 g, 2.9 equiv, 69.8 mmol), freshly distilled triethylamine (21.2 g, 3 equiv, 209.3 mmol), trichloro­(methyl)­silane (9 g, 1 equiv, 69.8 mmol), and 450 mL of dry diethyl ether. Purification was also performed as described for TSE. The product was obtained as a clear oil (21.2 g, 74% yield). Purity was shown via UHPLC-MS (see SI).

^1^H NMR (400 MHz, CD_2_Cl_2_) δ 6.21–5.89 (m, 6H, -CH=CH-), 3.87–3.21 (m, 6H, -O-CH_2_-), 3.02–2.66 (m, 6H, -CH-CHR-CH=), 2.41–0.33 (m, 15H, aliphatic Hs), 0.19–0.02 (m, 3H, R_3_Si–CH_3_). ^13^C NMR (101 MHz, CD_2_Cl_2_) δ 137.47 (-CH=CH-), 137.13 (-CH=CH-), 136.98 (-CH=CH-), 132.82 (-CH=CH-), 67.20 (-O-CH_2_-), 66.42 (-O-CH_2_-), 49.71 (bridging carbon -CH-CH_2_–CH-), 45.15 (bridging carbon −CH-CH_2_–CH-), 44.14 (-OCH_2_–CH-), 43.78 (-OCH_2_–CH-), 42.74 (-CH-CHR-CH=), 42.03 (-CH-CHR-CH=), 41.84 (-CH-CHR-CH=), 41.76 (-CH-CHR-CH=), 29.66 (-OCH_2_–CHR-CH_2_), 29.08 (-OCH_2_–CHR-CH_2_). ^29^Si NMR (80 MHz, CD_2_Cl_2_) δ −43.50. *R*_f_ (AlOx neutral, PE/EE = 20:1): 0.84.

### 2.6 Preparation of Formulations and Cured Polymer Specimens

Liquid formulations for determination of photoreactivity, 3D printing, and preparation of specimens for material testing (dynamic mechanical thermal analysis (DMTA), tensile tests, cytotoxicity evaluation, and degradation) were freshly prepared under exclusion of light wavelengths <480 nm. Pyrogallol was used at a concentration of 0.02 wt % for enhanced stability, and Ivocerin was used at a concentration of 1 wt % to enable light-induced polymerization (wt % of total formulation). Monomers were used in a 1:1 thiol/ene ratio, and their mass fraction corresponded to 98.98 wt % of the total formulation mass. To ensure sufficient homogenization, a vortex and slight heating were employed. Specimens for material testing were prepared via casting in respective silicone molds. Photocuring was conducted at rt using a Lumamat 100 light oven equipped with six Osram Dulux L Blue 18 W lamps (400–580 nm). Specimens were cured for 10 min on each side, followed by a thermal postcuring step at 100 °C overnight. DMTA and tensile test specimens were sanded to provide a defect-free surface.

### 2.7 Characterization

For photorheological analysis, an Anton-Paar Modular Compact Rheometer MCR 302 WESP was used with an Omnicure S2000-XLA, equipped with a filter to only let wavelengths between 400 and 500 nm pass, as a light source. Light intensity, which was set at 40 mW cm^–2^, was determined with the help of a USB2000+ radiometer from Oceanoptics. Measurements were performed with a 65 s blank period in the oscillation mode before irradiation started. After that, irradiation was performed for 540 s in total while data points were taken every 0.2 s for the first 300 s and every second for the following 240 s. Five seconds prior to irradiation, NIR spectra acquisition started. For this, an FTIR spectrometer (Bruker Vertex 80) and a mercury–cadmium–telluride (MCT) detector were used. For further details on the experimental setup and data analysis, reference is made to a publication of Gorsche et al.^40^

Attenuated total reflectance–Fourier transform infrared (ATR-FTIR) spectroscopy was performed by using a PerkinElmer Spectrum 65 FT-IR spectrometer equipped with a Specac MKII Golden Gate Single Reflection ATR System. For quantitative analysis, the C–H stretching band from 3035 to 2790 cm^–1^ was used as reference. Conversion was calculated with the alkene C–H out of plane bending peak for norbornene functionalities at 735–695 cm^–1^ and the alkene C–H stretch peak for vinyl groups at 1655–1625 cm^–1^. Measurements were performed in triplicate.

Dynamic mechanical thermal analysis (DMTA) was performed with an Anton Paar MCR 301 device with a CTD 450 oven and an SRF 12 measuring system in torsion mode. The temperature range was from −100 to 200 °C with a heating rate of 2 °C min^–1^. The strain was 0.1%, and frequency was 1 Hz. DMTA specimens with dimensions of 1 × 5 × 40 mm^3^ were used. The glass transition temperature, *T*_g_, was calculated using the RheoCompass software by Anton Paar and the half-step height method similar to DIN EN ISO 11357-1 and DIN EN ISO 11357-2.

Tensile tests were performed on a Zwick Z050 tensile testing machine equipped with a 1 kN load sensor. Bone-shaped specimens with shape 5B according to ISO527-2:2019 were fixated with two clamps before strain application (5 mm min^–1^). The resulting stress–strain curve gives information about the ultimate tensile strength (σ_M_), elongation at break (ε_B_), the tensile toughness (*U*_T_), and the slope of the linear region of the stress–strain curves, indicating the materials’ stiffness.

Cytotoxicity was assessed by quantifying the viability of NCTC 929 mouse fibroblast cells exposed to various NM-dimer concentrations after 24 h. The fibroblasts were cultured in Dulbecco’s Modified Eagle Medium (DMEM) supplemented with 10% fetal bovine serum (FBS) and 1% antibiotic–antimycotic solution at 37 °C and 5% CO_2_, used in the experiment between passages 7 and 8, and maintained by standard cell culture techniques. Culture medium was replaced every 2 days, and cells were subcultivated twice a week at a 1:5 seeding ratio, performing cell detachment via trypsinization. On day 0, cells were seeded into the respective wells (5000 cells/well) of a 96-well plate and incubated for 24 h in complete medium (with 10% FBS and 1% antibiotic–antimycotic solution). On day 1, a 1 M solution of NM-dimer in DMSO was added in the required amounts to DMEM without supplementation of FBS and antibiotics for final concentrations of 2.5, 2, 1, 0.5, and 0.1 mM. The dilutions were sterile-filtered, applied to the wells (*n* = 8 biological replicates), and incubated for 24 h. Control groups such as live control (untreated, only DMEM), dead control (10% Triton X in PBS), and DMSO (same amount as for 2.5 mM NM-dimer dilution) were introduced to validate the PrestoBlue cytotoxicity assay on day 2 after 24 h of exposure. In brief, cells were washed twice with DMEM without supplementation of FBS and antibiotics before the addition of 100 μL of PrestoBlue solution diluted 1:10 in DMEM and incubated for 1 h at 37 °C, 5% CO_2_. Fluorescence was measured at an excitation wavelength of 560 nm and emission wavelength of 590 nm with an EnSpire Plate reader (PerkinElmer). Background was subtracted (DMEM) and data were normalized to live control (100%).

Hydrolytic degradation was investigated with cylindrical samples (diameter = 5 mm). They were immersed in respective buffer solutions (∼15 mL per sample) for 2, 7, 14, 30, 61, 90, and 180 days at 37 °C. Three different buffers were used: acetate buffer (pH 4), phosphate-buffered saline (pH 7.4), and carbonate buffer (pH 10). At each time point, the pH values were checked with a Mettler Toledo Seven2Go pH meter S2 equipped with an InLab Micro Pro-ISM pH electrode. Samples were blot dried one time on each side in order to assess the swollen mass and rinsed with deionized water, and after that they were dried to constant weight at 50 °C and weighed again.

### 2.8 Additive Manufacturing

Printing was conducted on a DLP prototype printer with an In-Vision Ikarus 2 light engine (maximum light intensity 75 mW cm^–2^). The prototype printer was specially fabricated for small printing volumes with a maximum resin volume of 7.5 mL and a building platform size of 42 mm × 38 mm. For printing, a light intensity of 50 mW cm^–2^ and irradiation time of 4 s was set. Layer thickness was 25 μm. Printed parts were washed in acetone in an ultrasonic bath and postcured in a PrograPrint Cure from Ivoclar with 460 nm wavelength and a light intensity of 274 mW cm^–2^ for 10 min.

## 3 Results and Discussion

To show the positive influence of norbornane motifs in a polymeric network structure, a trifunctional silyl ether-based monomer containing norbornene side groups, termed NSE, was envisioned (Scheme 1). As norbornenes are known to be highly reactive toward radical thiol–ene click chemistry, a respective norbornane-containing a thiol comonomer, termed TSE, was envisioned as well.^41^ Besides being a fast and atom economic polymerization method, radical thiol–ene polymerization also allows the fabrication of highly homogeneous networks with sharp glass transitions, which is also desirable for medical use.^41–44^

**Scheme 1:**
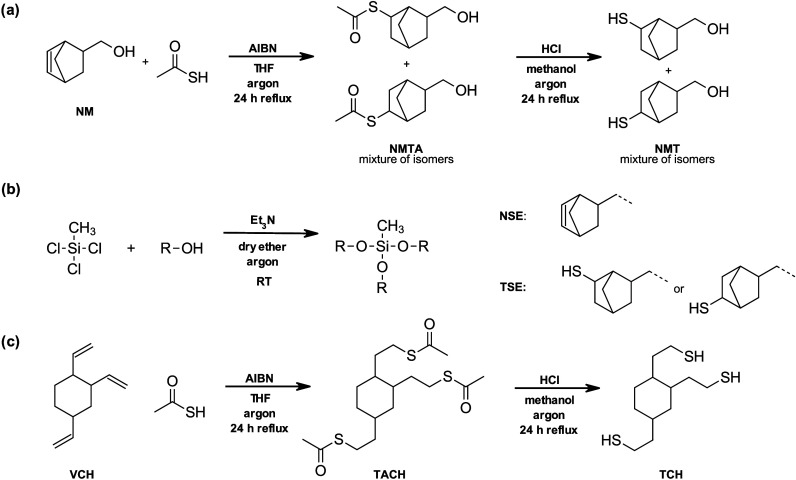
(a) Synthesis of Monomer Precursor NMT; (b) Synthesis of Silyl Ether-Based Monomers NSE and TSE; (c) Synthesis of Noncleavable Monomer TCH

### 3.1 Synthesis of Monomers and Network Design

To demonstrate the enhanced mechanical properties that came with the introduction of norbornene-based side groups, two model monomers, one double bond containing monomer and one thiol monomer, were synthesized starting from (5-norbornene-2-yl)­methanol (NM) and trichloro­(methyl)­silane. The norbornene silyl ether monomer, NSE, with ene moieties was prepared directly from NM and trichloro­(methyl)­silane in a nucleophilic substitution with triethylamine as acid scavenger in a one-step reaction adapted from Ware et al. (see Scheme 1b).^23^ The synthesis of the thiol silyl ether monomer, TSE, included the preparation of the monomer precursor NMT, which was synthesized in a two-step process (Scheme 1a). Briefly, NM was reacted with thioacetic acid by means of a thiol–ene click reaction in THF following a protocol by Reinelt et al., giving NMTA in a mixture of isomers.^39^ To obtain the free thiol NMT, NMTA underwent acidic hydrolysis in methanol under oxygen-free atmosphere (i.e., argon atmosphere) to avoid disulfide-bond formation. NMT was received in high yield and was further reacted with trichloro­(methyl)­silane, similarly to the synthesis of NSE, to give the thiol silyl ether TSE. Both monomers, NSE and TSE, were purified via vacuum column chromatography over neutral aluminum oxide and were obtained in reasonable yields.

To be able to fully investigate both silyl ether monomers, NSE and TSE, two noncleavable, literature-known reference compounds were chosen as comonomers: the commercially available cyclohexane-based vinyl monomer VCH and the synthesized thiol monomer TCH introduced by Van Damme et al. (see Figure 1).^45^ The synthesis of TCH (for experimental details see SI) was performed similarly to that for NMT and was adapted from Reinelt et al. as well (see Scheme 1c).^39^

**Figure 1:**
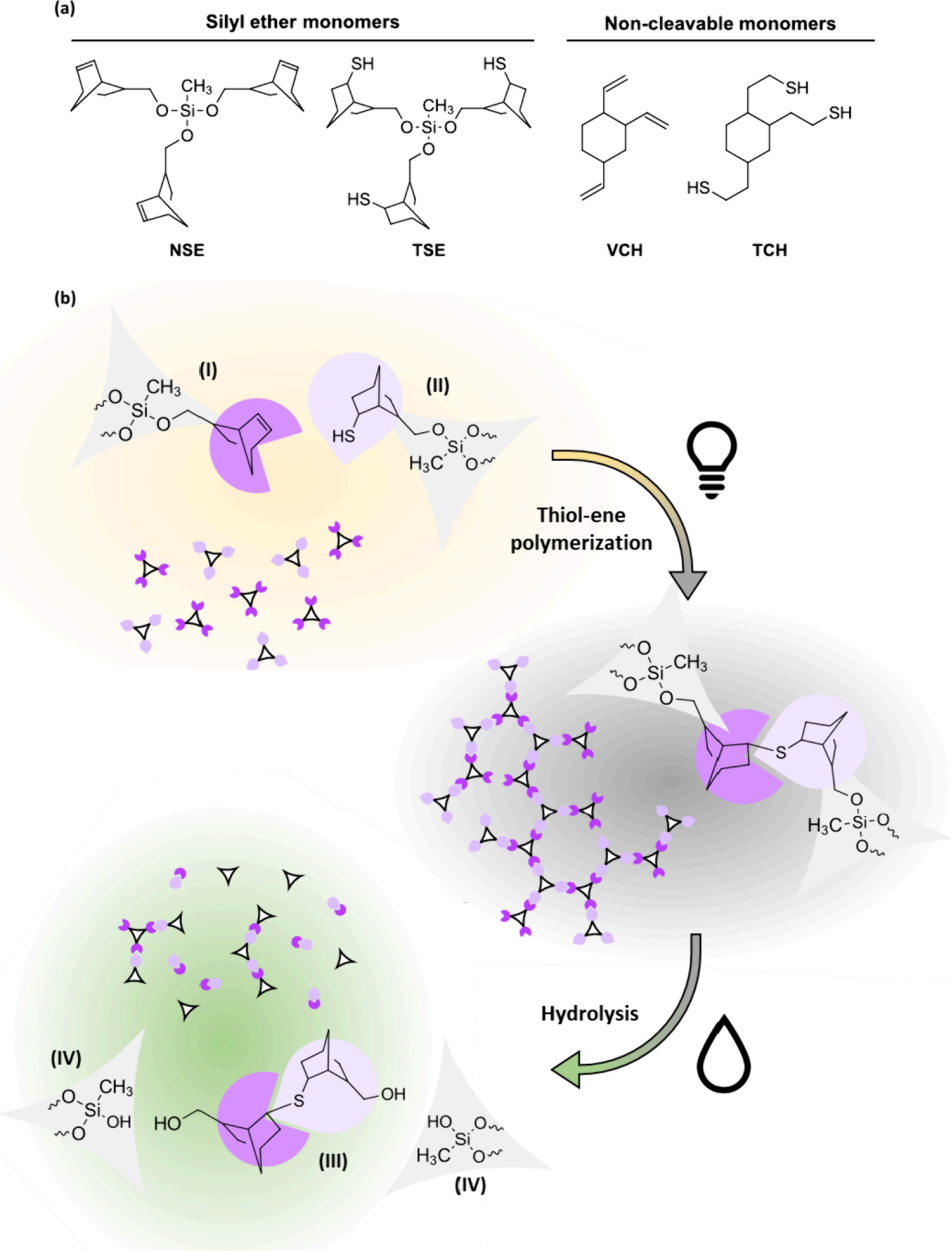
(a) Structures of the silyl ether-based ene monomer NSE, the silyl ether-based thiol monomer TSE, the noncleavable commercially available ene monomer VCH, and the synthesized noncleavable thiol monomer TCH. (b) Schematic depiction of network formation with NSE (I) and TSE (II) as monomers via thiol–ene photopolymerization and network degradation via hydrolytic cleavage of Si–O bonds with the resulting degradation products, NM-dimer (III) and silanol species (IV).

In order to obtain a photocurable formulation for subsequent thiol–ene photopolymerization (see Figure 1b), ene and thiol monomers had to be combined with a photoinitiator. To ensure proper step-growth polymerization, ene and thiol monomers were mixed in a molar 1:1 ratio under addition of 1 wt % Ivocerin as photoinitiator and 0.02 wt % pyrogallol as stabilizer. For a thorough investigation, each ene monomer was combined with each thiol monomer. This resulted in a degradable network termed NSE-TSE, two mixed networks (NSE-TCH and VCH-TSE) and one nondegradable network VCH-TCH, whereby both mixed networks, in theory, would be chemically identical. The structures of used monomers can be found in Figure 1a. To avoid premature gelation, the photoinitiator and stabilizer were dissolved in the ene monomer first during preparation of the formulation. Following this, the thiol monomer was added. The resulting homogenized liquid formulation was used directly for further characterization. All formulations were fully miscible at room temperature and therefore further investigated under ambient conditions. Cured networks for the assessment of material properties were obtained by casting into respective silicone molds and subsequent photocuring and thermal postcuring.

### 3.2 Photoreactivity

As the favored fabrication technique for bone scaffold materials is SLA printing, one crucial parameter is the photoreactivity of the newly synthesized monomers. A powerful tool for assessment of a formulations’ photoreactivity is real-time near-infrared (RT-NIR) photorheology.^40^ With this technique, mechanical and chemical data (i.e., conversion of double bonds) can be acquired simultaneously. The conversion of the double bonds was representative for overall monomer conversion in this study, as both ene monomers, NSE (norbornene groups) and VCH (terminal alkene groups), do not undergo homopolymerization. Steinbauer et al. demonstrated that no norbornene homopolymerization after UV irradiation takes place in the presence of Ivocerin as photoinitiator.^46^ In a publication by Hoyle and co-workers, discussing alkene reactivity in thiol–ene click reactions, they outlined that simple alkenes do not undergo homopolymerization.^47^

A fast gel point, *t*_gel_ (defined as the intersection of the storage modulus, *G*′, and the loss modulus, *G*″) with high conversions should be targeted to enable a fast printing job resulting in robust materials. Furthermore, reaction kinetics should not be underestimated, as they play a decisive role in the final mechanical properties of printed parts. Formulations that reach their *t*_gel_ at a very early stage of the polymerization reaction (i.e., at low double bond conversion, DBC), typically tend to develop higher stress within the material. This so-called shrinkage stress could lead to material failure, for example, to delamination during 3D-printing.^48^

High reactivity and fast gelation were observed for the degradable network, NSE-TSE, and the nondegradable network, VCH-TCH. The *t*_gel_ for both formulations was ∼2 s, and both also exhibited already high values for DBC_gel_. By virtue of these findings, NSE-TSE and VCH-TCH obtained sufficient reactivity for SLA printing. Investigation of the mixed networks NSE-TCH and VCH-TSE revealed that their behaviors upon irradiation were quite different. The reason for this could be easily explained by the highly different reactivities of the functional groups participating in the thiol–ene click reaction. The reactivity of different ene and thiol monomers was extensively studied and discussed in literature.^41,49,50^ The norbornene silyl ether monomer, NSE, comprised three norbornene moieties, which are reported to be highly accessible via radical thiol–ene reactions due to release of ring strain during conversion.^41^ In contrast to this stands the thiol silyl ether monomer, TSE, possessing three secondary thiol groups. Long et al. demonstrated that thiol reactivity for radical thiol–ene reactions decreased with increasing degree of substitution at the α-carbon most likely due to steric effects.^51^ According to those findings, the introduction of NSE or TSE, respectively, to a formulation with good photoreactivity should lead to an increase in reactivity with NSE and a decrease with TSE. This exact behavior is displayed in Figure 2. The mixed network NSE-TCH gelled already after 2.5 s, achieved 95% of its final DBC after only 5 s, and achieved quantitative conversion in the end. The second mixed network, VCH-TSE, on the other hand, cured rather slowly, exhibited a *t*_gel_ of 17 s, and was therefore less attractive for SLA printing. However, although both mixed systems differed in their reactivity, their DBC_gel_ was rather high, indicating lower shrinkage stress within the final material.^48^ As the gap between the final DBCs of the mixed networks was ∼35%, differences in the mechanical behaviors could be expected. Even though the chemical composition of the networks was the same, the difference in the final DBC resulted in a fairly distinct cross-linking density.

**Figure 2:**
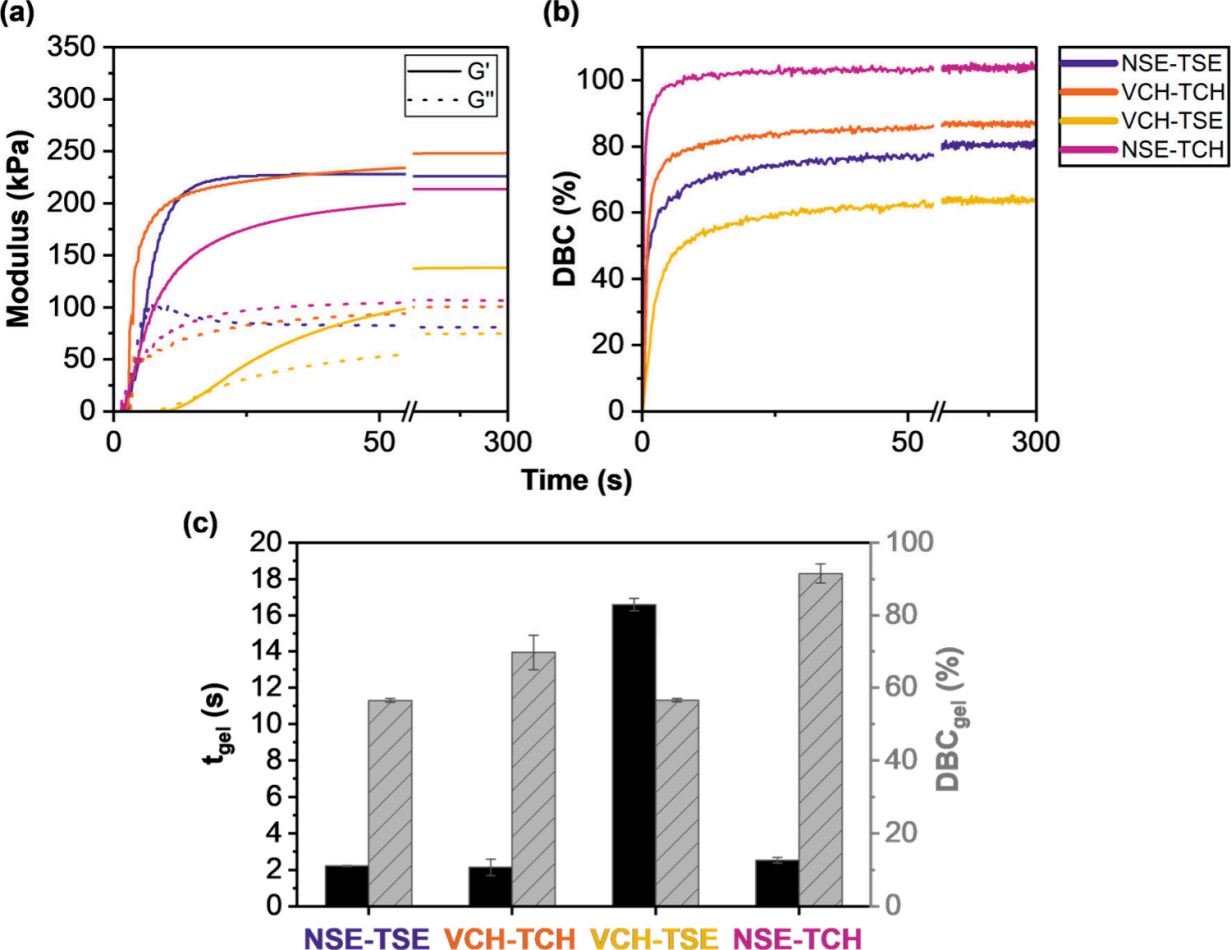
(a) Storage and loss modulus (kPa) of formulations during irradiation (started at *t* = 0 s, 40 mW cm^–2^) containing silyl ether monomers (NSE and TSE) and nondegradable monomers (VCH and TCH) in an equimolar thiol-to-ene ratio, 0.02 wt % pyrogallol as inhibitor, and 1 wt % Ivocerin as photoinitiator. (b) Double bond conversion (%) measured via real-time near-infrared spectroscopy during irradiation. (c) Gel points, *t*_gel_, and double bond conversions at the gel point, DBC_gel_, of tested formulations.

Similar trends were shown during the ATR-FTIR spectroscopy assessment of specimens cured in bulk. Spectral acquisition of uncured formulations and photocured (irradiation only) and postcured (irradiation +100 °C overnight) parts allowed comparison of spectral bands and calculation of the DBC under consideration of a reference band. Also here, the mixed network NSE-TCH showed the highest initial conversion at 87% (±1%), but also the highest conversion after thermal treatment at 98% (±1%). This was followed by the degradable network NSE-TSE (84% ± 2% initial, 97% ± 2% postcured). The mixed network VCH-TSE (78% ± 1% initial, 82% ± 1% postcured) and the nondegradable network VCH-TCH (77% ± 2% initial, 79% ± 1% postcured) showed results in a similar range. As demonstrated above, there are major differences in the final DBCs of the mixed networks. After postcuring, there was still a difference of 17% DBC found for NSE-TCH and VCH-TSE. Data acquired during this bulk material investigation support the general trends regarding the behavior of the mixed networks that were observed during RT-NIR photorheology experiments.

### 3.3 (Thermo)­mechanical Properties

To provide sufficient temporal support as a bone graft, the (thermo)­mechanical properties of material candidates are paramount. Besides exhibiting adequate mechanical strength, materials for bone regeneration should also properly perform at body temperature. Therefore, it is crucial to carefully investigate temperature-dependent material behavior, which was done by employing dynamic mechanical thermal analysis (DMTA). Response toward tensile load at rt was tested with tensile test experiments. Numeric data can be found in Table 1.

**Table 1:** Glass Transition Temperature, *T*_g_ (°C), Storage Modulus at 37 °C, *G*_37°C_^′^ (MPa), and Tensile Strength at rt (MPa) of Cured Polymer Networks Containing Silyl Ether Monomers (NSE and TSE) and Nondegradable Monomers (VCH and TCH)

	*T*_g_ (°C)	*G*_37°C_^′^ (MPa)	tensile strength (MPa)
NSE-TSE	62	1215	34.9 ± 4.5
VCH-TCH	36	50.4	28.3 ± 0.9
VCH-TSE	17		2.8 ± 0.3
NSE-TCH	19		52.7 ± 1.1

The nondegradable network composed of the monomers VCH and TCH (solid yellow line in Figure 3) exhibited a high rubbery plateau during DMTA, which indicated the highest cross-linking density compared to the other tested polymer networks. Switching one of the monomers to a degradable one (NSE or TSE) results, at least in theory, in two polymeric networks comprising the same network architecture. Consequently, those networks should also display similar (thermo)­mechanical behaviors. The curves in Figure 3 show this exact phenomenon. For both mixed networks (dashed-dotted lines), VCH-TSE (orange) and NSE-TCH (pink), the storage modulus *G*′ started to decrease around 5 °C, followed by a rapid and sharp drop of the modulus to the rubbery plateau. The range of declining *G*′ for the mixed networks is magnified in Figure 3a for better visibility. The very sharp phase transition was also clearly observable in the loss factor curve. This sharpness implied a high network homogeneity for both mixed polymer networks. Observed *T*_g_ values were rather low for both networks: 17 °C for VCH-TSE and 19 °C for NSE-TCH. Hence, both mixed networks were found to be unsuitable for bone tissue engineering (BTE) applications. Until now, former literature suggested that with increasing content of silyl ether monomer, the *T*_g_ was lowered due to the high flexibility of the Si–O bond.^24^ However, when the degradable network NSE-TSE (dotted line), which exhibited the highest norbornane motif content, was considered, the trend formerly described was not followed. Compared to the mixed networks, the *T*_g_ of the silyl ether network NSE-TSE (62 °C) was more than tripled and was therefore sufficiently higher than body temperature. Along with this came a significant increase in the storage modulus at 37 °C (*G*_37°C_^′^), an indicator for material stiffness, which was measured to be 1215 MPa.

**Figure 3:**
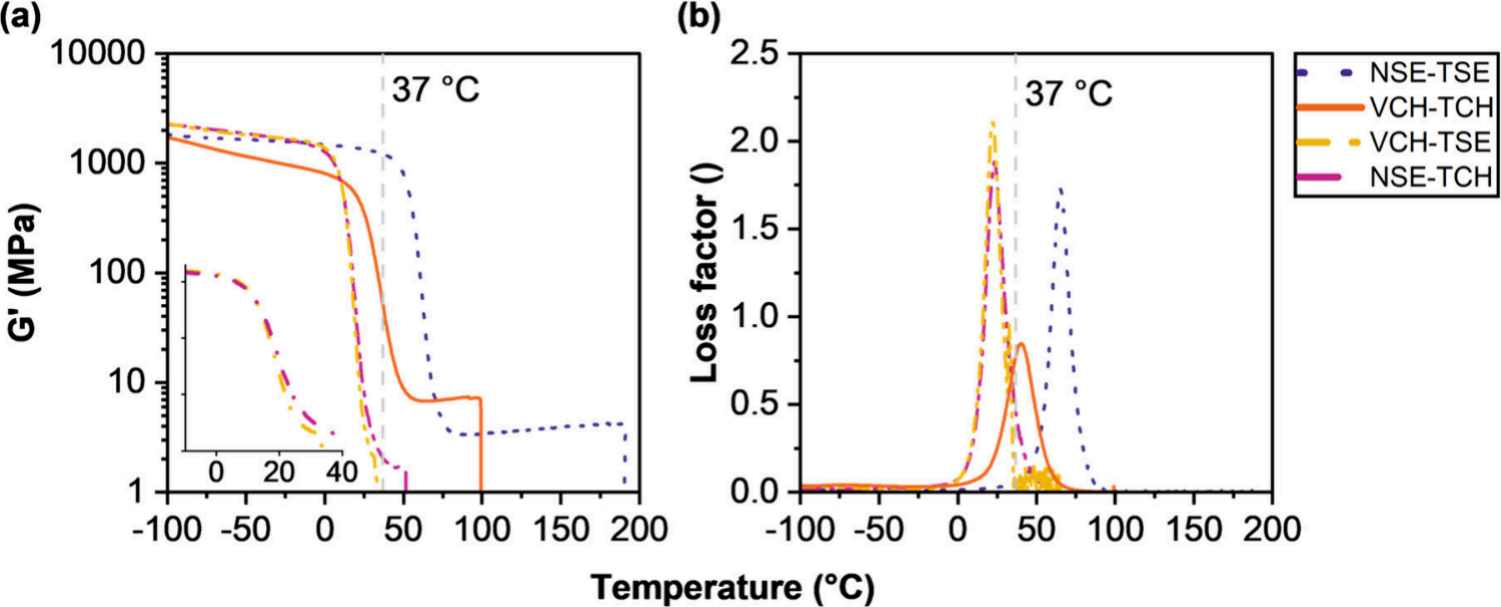
(a) Storage modulus, *G*′ (MPa), and (b) loss factor (unitless) of cured polymer networks containing silyl ether monomers (NSE and TSE) and nondegradable monomers (VCH and TCH), 0.02 wt % pyrogallol, and 1 wt % Ivocerin. Abrupt drops of *G*′ can be attributed to slipping of the specimens from the fixation clamps caused by the high elasticity of the materials after reaching their glass transition temperature. (a) Inset: magnification of glass transition of the cured polymer networks VCH-TSE and NSE-TCH for better visibility.

A materials’ response to mechanical load is also of utmost importance for BTE. The polymer networks were tested using a tensile test setup at rt to look into crucial parameters like tensile strength (σ_M_), elongation at break (ε_B_), and tensile toughness (*U*_T_). The slope of the stress–strain curve in its linear region was used for the evaluation of the materials’ stiffness, as no extensometer was used during tensile testing (detailed information in SI). As displayed in Figure 4, very favorable mechanical behavior was found for the nondegradable reference material VCH-TCH. The stress–strain curve showed a concise yield point (∼29 MPa) and a broad range of plastic deformation from approximately 6 to 40% strain. Strain hardening could be observed toward the end of the curve, which peaked in a tensile strength of 28 MPa. Although both mixed networks, VCH-TSE and NSE-TCH, demonstrated very similar temperature-dependent behavior during DMTA, they heavily differed in their performance when mechanical properties were investigated. Whereas NSE-TCH was shown to be a very stiff (slope = 1360 MPa) and mechanically strong material (σ_M_ = 53 MPa), its network analogue, VCH-TSE, displayed very high elasticity with a slope of 3 MPa and low tensile strength (2.8 MPa). The reason for this lies in the differences in monomer reactivity and conversion as secondary thiols are known to be less reactive in terms of thiol–ene chemistry.^51^ As described above, the difference in the final DBCs of the mixed networks resulted in a significant difference in their cross-linking density, which was showcased for thin layers during RT-NIR photorheology experiments, but also for bulk cured parts in ATR-FTIR spectroscopy measurements. Another good indicator for cross-linking density is the final *G*′ (see Figure 2).^52^ *G*_final_^′^ for NSE-TCH was relatively high at 203 kPa, while VCH-TSE only reached approximately two-thirds of this value (138 kPa), indicating a rather wide-meshed network.

**Figure 4:**
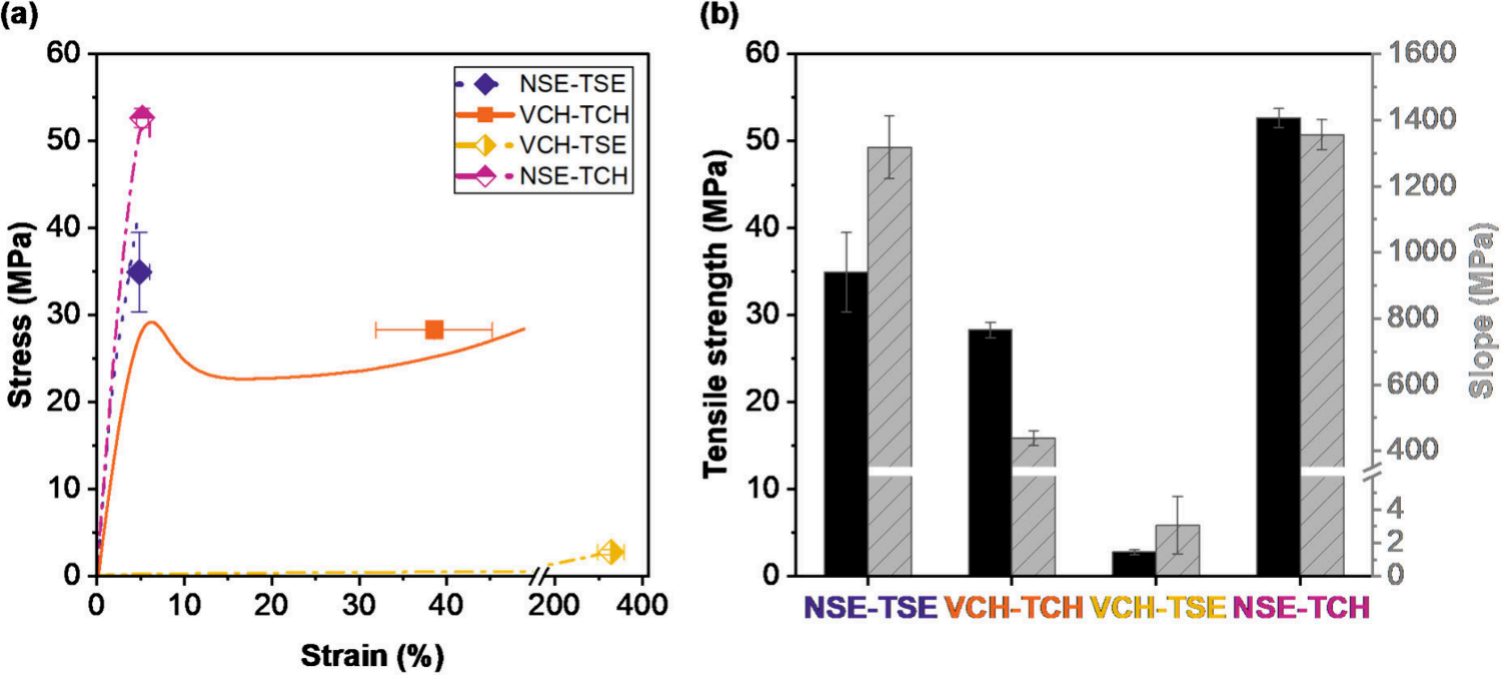
(a) Stress–strain curves obtained via tensile testing of cured polymer networks containing silyl ether monomers (NSE and TSE) and nondegradable monomers (VCH and TCH). (b) Tensile strength, σ_M_ (MPa, left *Y*-axis, black), and slope of the stress–strain curve in its linear region (MPa, right *Y*-axis, patterned gray) of cured polymer networks containing silyl ether monomers (NSE and TSE) and nondegradable monomers (VCH and TCH).

The cleavable, solely silyl ether-based network NSE-TSE displayed outstanding performance considering that this network was exclusively built from silyl ether-based monomers linked via thioether bonds. NSE-TSE even reached a higher maximum tensile strength than the nondegradable polymer network (28 vs 35 MPa). The slope of the linear region of the tensile test curve of the degradable network (1320 MPa) was comparable to that of the mixed network NSE-TCH (1360 MPa) and indicated a stiff and rigid polymeric material.

Overall, the silyl ether-based network NSE-TSE exhibited auspicious results. Besides a high *T*_g_ of 62 °C, the material was also found to be stiff and rigid and of high tensile strength at 35 MPa. With this, the degradable network NSE-TSE showed performance that was comparable to state-of-the-art thermoplastic polymers considered for BTE, such as PCL (σ_M_ = 25–43 MPa) or PLA (σ_M_ = 48–53 MPa).^14,53^ These properties and the sufficient formulation reactivity toward photopolymerization make NSE-TSE a promising candidate for SLA-fabricated bone scaffolds.

### 3.4 Degradability of Polymer Networks

Materials for bone grafting need to meet certain requirements, with one of them being sufficient degradability. The speed of the polymer network breakdown should roughly match the rate of bone formation.^9,54^

To demonstrate that the included Si–O bonds in the monomer structure are hydrolytically cleavable, hydrolytic degradation of the polymer networks was studied at different pHs (4, 7.4, and 10; see SI). Testing was performed in triplicate. The results for pH 7.4 are shown in Figure 5.

**Figure 5:**
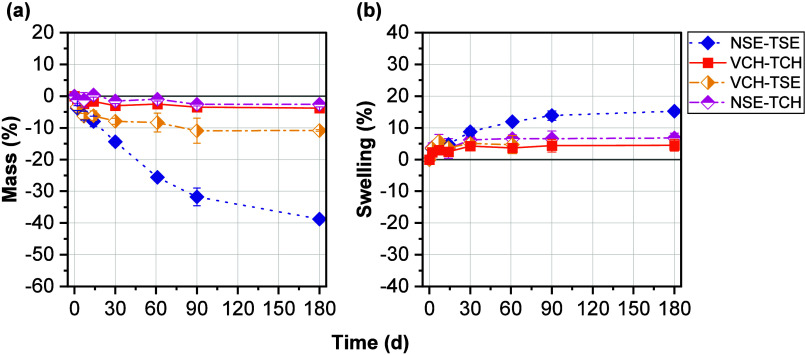
(a) Dry mass loss (%) of cured polymer networks containing silyl ether monomers (NSE and TSE) and nondegradable monomers (VCH and TCH) after storage at pH 7.4 and 37 °C. (b) Observed swelling (%) with respect to found dry masses at each time point.

The nondegradable material VCH-TCH did not show any substantial mass changes over the whole observation period. The mixed network with the silyl ether-based ene monomer NSE exhibited a similar behavior: there was almost no change in wet or dry mass. This was also true for pH 4 and 10 (see SI). One explanation for this may be the very high hydrophobicity of the NSE-TCH network and the high cross-link density measured in RT-NIR photorheology, both of which could negatively impact a polymers’ hydrolytic degradability.^54^ The mixed network analogue VCH-TSE showed improved degradation behavior with a final mass loss of 11% after 180 days at a pH of 7.4. However, during the last 3 months of the observation period, hydrolytic degradation stagnated as the final mass loss of 11% was also already found 90 days after the start of the experiment. Similar behavior was found at different pHs, except that after storage at pH 4, the final mass loss was higher at 20%. Swelling assessment was not possible after certain time points for pH 4 (after 61 days) and pH 7.4 (after 90 days) due to formation of a viscous layer that deposited on the surface of the sample, which most likely consisted of semidegraded, insoluble polymer. This layer may also have inhibited proper diffusion of buffer and degradation products and slowed degradation with time.

The fastest degradation was observed for the degradable network NSE-TSE. The final mass loss was 39%, resulting in an average degradation rate of 6.5% per month. Mass loss took place in a linear fashion and swelling settled at approximately 15% after 90 days, which indicated a surface erosion mechanism.^8^ With the swelling ratio being only 10% higher than for the nondegradable reference VCH-TCH, it was still within an acceptable range. At decreased pH, faster degradation and substantial swelling (57%) were observed (see SI).

Overall, mass loss and moderate swelling were witnessed for the degradable network NSE-TSE and the mixed network VCH-TSE during storage at a pH of 7.4, although, for the latter, degradation seemed to stagnate after 90 days.

### 3.5 Cytotoxicity of Network Degradation Products

Since the degradable network NSE-TSE showed proper photoreactivity, (thermo)­mechanical properties, and degradation behavior for 3D-printed bone scaffold materials, the next crucial aspect to elucidate was the biocompatibility. Toxicity of polymeric materials, be it thermoplasts or thermosets, is mainly associated with the leaching of unreacted monomers, solvents used for manufacturing, initiators, additives, etc.^24,55^ Therefore, the first step should be the assessment of the unreacted monomers. However, after investigating monomer solubility by preparation of a 1 M solution of the respective monomers in DMSO for subsequent dilution with respective amounts of cell culture medium, it was found that the silyl ether-containing monomers did not even dissolve in pure DMSO. The silyl ether-containing ene monomer NSE formed a biphasic mixture with DMSO, while the mixing of the corresponding thiol TSE with DMSO led to a turbid liquid, even at elevated temperatures of 37 °C. Hence, a cytotoxicity study of unreacted monomers was not carried out (see SI). The used photoinitiator Ivocerin is a widely used component in biomedical applications and was hence also excluded from cytotoxicity testing as there is existing data in this regard.^20,56,57^ The stabilizer pyrogallol was used as a state-of-the-art compound to enhance thiol–ene resin stability.^58^ However, it is known that pyrogallol can be problematic when administered in high dosages, but there are alternatives available.^59,60^ He and co-workers demonstrated the use of naturally occurring polyphenols, e.g., gallic acid and tannic acid, as radical inhibitors for photocurable resins.^61^ While tannic acid is a component often used in biomedical engineering, gallic acid is reported to have cytotoxic effects toward certain cancer cells without affecting normal cells.^62,63^ Therefore, both substances could be considered as a biocompatible alternative for pyrogallol.

One class of chemical compounds could have significant influence on the biocompatibility of silyl ether-based polymer networks: the degradation products. To investigate the biocompatibility of the resulting degradation products, it is important to understand the degradation mechanism and the respective products that result from network cleavage (see Figure 1b). During network degradation, water attacks the silicon atom of the labile silyl ether bond. This leads to a scission of the Si–O bond under the formation of a silanol and an alcohol. Regarding the bioactivity of silanol groups, there are several studies implying beneficial effects of Si–OH groups for bone growth and mineralization.^64–66^ Hence, the one remaining compound to be investigated regarding cytotoxicity was the diol degradation product termed the NM-dimer (see Figure 1b­(III)). For this, the NM-dimer was synthesized and purified using column chromatography (see SI). The purity was shown via UHPLC-MS.

To assess the influence of the NM-dimer on cell viability, the compound was tested in various concentrations with NCTC clone 2959 mouse fibroblasts in a PrestoBlue assay. For this, cultivated cells (5000 cells/well) were incubated for 24 h in Dulbecco’s Modified Eagle Medium (DMEM) without supplements, spiked with the respective amounts of 1 M NM-dimer solution in DMSO to achieve the needed concentrations. For better comparability, a DMSO control group (same amount of DMSO as used for 2.5 mM NM-dimer group) was included in the study as well.

Figure 6a shows the cell viability of different groups, with the live control viability being set to 100%. As anticipated, DMSO addition seemed to have a minor effect on cell viability, comparable to the effect of the 0.1 mM NM-dimer. In contrast, the highest tested NM-dimer concentration of 2.5 mM already decreased cell viability to 20%. The results found in this assay were also supported by microscopy (see SI). For the calculation of LC_50_, the concentration that is lethal to 50% of the cell population, the found viabilities were plotted against the respective concentrations. With this, it was possible to interpolate LC_50_, which was found to be 1.45 mM. Comparing this to toxicities of degradation products of poly­(lactic acid) at 0.1 mM or of poly­(ε-caprolactone) at 0.12 mM, both of which are among the most commonly used polymers for biomaterials, the found LC_50_ of 1.45 mM indicated better performance of the solely silyl ether-based networks in terms of cytocompatibility.^67,68^ Assuming the average blood volume of 5 L for an adult human, 7.25 mmol of NM-dimer would have to be set free at once in order to reach LC_50_.^69^ This corresponds to 2.2 g of cross-linked NSE-TSE material, which would have to degrade within a short period spontaneously. Considering the observed average degradation rate per day under physiological conditions (∼0.22%), the maximum implant weight should not exceed ∼1.02 kg, of course neglecting numerous factors like surface-to-volume ratio, which heavily influences the rate of degradation or differences between static and dynamic environments.^9^ These numbers should, of course, be seen only as estimates. In the end, it was found that the degradation product of the cleavable silyl ether network NSE-TSE exhibited very promising cytocompatibility with mouse fibroblast cells.

**Figure 6:**
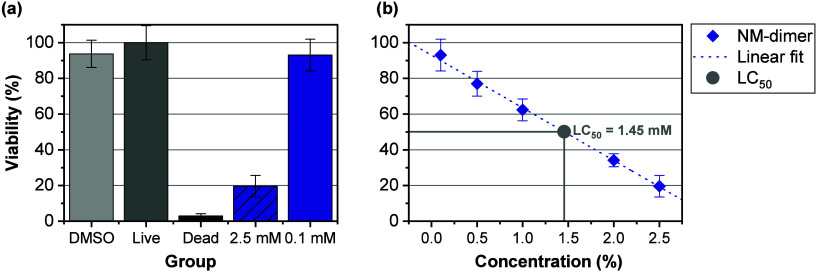
Viability of NCTC clone 929 mouse fibroblast cells (%) after 24 h of incubation with different concentrations of NM-dimer in DMEM via PrestoBlue assay (*n* = 8 biological replicates). Live control was set to be 100% viability. (a) Viability (%) after 24 h incubation under different conditions. DMSO (conc. same as for 2.5 mM NM-dimer medium), Live (untreated cell culture), and Dead (cell culture treated with 10% Triton X for 30 min) were used as control groups. The highest tested NM-dimer concentration (2.5 mM) and the lowest tested concentration (0.1 mM) are compared. (b) Linear fit of viability depending on NM-dimer concentration (*R* ^2^ = 0.998) and calculated LC_50_.

### 3.6 Additive Manufacturing

SLA printing offers many opportunities for tissue regeneration, such as fast and economic production of patient-specific implants with sufficient porosity to enable nutrient and waste product transport, vascularization, etc.^4,70^ Based on the results regarding fast gelation, good mechanical strength, and stability also at enhanced temperatures, the degradable silyl ether-based formulation containing the monomers NSE and TSE was chosen for 3D printing. Preliminary tests showed that no light absorbers were necessary to provide a high printing resolution. Therefore, the printing formulation did not contain any additional compounds other than pyrogallol as state-of-the-art stabilizer and Ivocerin as photoinitiator. The printing parameters for the following proof-of-concept prints were determined with the help of irradiation tests and generation of Jacobs’ working curve (see SI) as described in the literature.^71,72^ The printing experiments were performed on a DLP printer, which enabled fast and even curing for each printed layer. The light engine provided a wavelength of 385 nm. Printing was conducted with a light intensity of 50 mW cm^–2^ and an irradiation time of 4 s per layer, which resulted in a light dose of 200 mJ cm^–2^. According to the generated working curve, the theoretical curing depth (*C*_d_) for this light dose was 67 μm. To achieve a sufficient curing overlap between the layers, the layer thickness during printing was set at 25 μm. Appropriate flowability of the liquid formulation was ensured by slight increase of the vat and platform temperature to 35 °C.

A flat lattice structure (Figure 7a) was printed first in order to demonstrate the printability of delicate, porous structures. Microstructure investigation via SEM revealed good visibility of the individual voxels and very good resolution of each cavity (Figure 7c, 472 μm × 476 μm) compared to the digital file (Figure 7d, 500 × 500 μm) resulting in a discrepancy of ∼6%. To further demonstrate the achieved spatial resolution, a test chip with two converging areas was printed. According to the digital file, the void between the areas was set at 100 μm × 100 μm, i.e., 2 px × 2 px. The achieved dimensions of 92 μm × 85 μm for the void demonstrate good spatial resolution.

**Figure 7:**
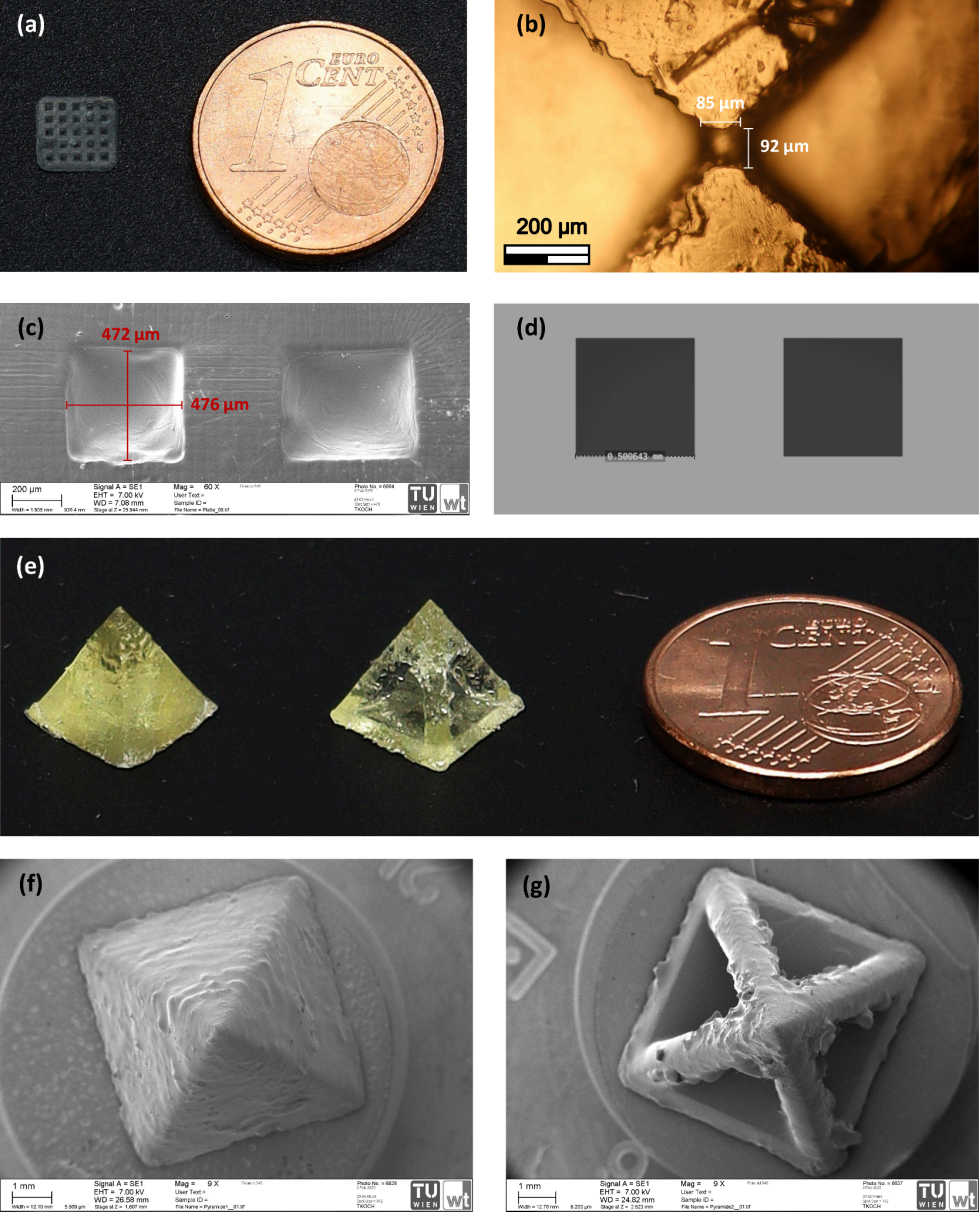
Images of 3D-printed parts. (a) Lattice structure (5 mm × 5 mm) with 5 × 5 cavities (500 μm × 500 μm/10 px × 10 px acc. to digital file). (b) Microscopy image of test chip for assessment of printing accuracy (100 μm × 100 μm/2 px × 2 px void acc. to digital file). (c) SEM image of lattice structure surface with visible voxels (500 μm × 500 μm/10 px × 10 px acc. to digital file). (d) Digital file of lattice structure with measured cavity size. (e) Full and hollow pyramid (7 mm × 7 mm × 7 mm). (f) SEM image of a full pyramid. (g) SEM image of a hollow pyramid.

To show that the fabrication of geometries with increased height was feasible, a full pyramid (7 mm × 7 mm × 7 mm) was printed as a proof-of-concept. The printed pyramid exhibited well-defined edges, but especially toward its top, a loss in printing accuracy was observed. This could be on the one hand attributed to overpolymerization, an effect that is caused by the diffusion of reactive species out of the irradiated area, and on the other hand, to premature thermal gelation of the nonirradiated formulation.^44^ As no delamination occurred until now, a hollow pyramid (7 mm × 7 mm × 7 mm) was printed to demonstrate sufficient interlaminar binding between the layers and adequate mechanical properties in order to print more challenging structures. The print resulted in a mechanically stable and stiff hollow pyramid. However, also here, a loss of printing accuracy was observed.

When a closer look at the hollow pyramid is taken, it can be hypothesized that this may be time-dependent. In SEM images it can be seen that the longer the print went on, the more overpolymerization/thermal gelation took place (see Figure 7g): the bottom platform of the hollow pyramid still showed sharp edges and a very smooth surface, while the pillars developed a more uneven surface toward the top. Since the *xy*-resolution of the lattice print was decent, it was concluded that the observed loss in accuracy was a consequence of long-time exposure to elevated temperature. To reduce this in future prints, either printing parameters could be optimized or further additives could be employed to enhance its temperature- and time-dependent stability.

## Conclusions

Stereolithography offers a promising opportunity for manufacturing of customized bone scaffolds. Until now, the material platform for this technique has been rather limited, as most of the state-of-the-art monomers are based on (meth)­acrylic systems, posing a cytotoxicity risk and insufficient degradation behavior. To expand the variety of degradable monomers that could be employed for SLA and to provide homogeneous photopolymers, silyl ether-based ene and thiol monomers were introduced for thiol–ene click photopolymerization. Silyl ethers are synthetically well accessible via nucleophilic substitution of chlorosilane and an alcohol. Due to their crucial role in the silicon industry, chlorosilanes are widely available and cheap, as they are produced in large amounts in the Müller–Rochow process.^73^

To overcome the rather weak (thermo)­mechanical properties that come with the employment of Si–O bonds, rigid norbornane moieties were introduced into the polymer networks. The silyl ether-based norbornene monomer, NSE, could be prepared in a facile one-step reaction, while its thiol analogue, TSE, was successfully synthesized in a three-step procedure. Combination with noncleavable comonomers VCH and TCH resulted in a wide variety of mechanical properties, which provides tunability of material characteristics. For the solely silyl ether-based network NSE-TSE, fast gelation was found, which enables fast 3D structuring as well as an excellent *T*_g_ of 62 °C and high mechanical strength. As mentioned above, the maximum tensile strength of NSE-TSE was comparable to PCL or PLA and lies between the tensile strengths of cortical (50–150 MPa) and cancellous bone (10–20 MPa).^4^ One possibility to better mimic endogenous bone in terms of mechanical properties is, e.g., the introduction of particle fillers. Hydrolytic degradation was displayed at a rate of ∼6.5% mass loss per month during storage at physiological conditions, and the resulting diol degradation product did not show substantial toxicity for mouse fibroblast cells after exposure for 24 h. In the end, NSE-TSE was successfully printed in various proof-of-concept structures, including lattice structures to demonstrate the printability of pores and full and hollow pyramids that showed sufficient mechanical stability of the printed parts. With this, the silyl ether-based degradable photopolymer network NSE-TSE offers great potential for future bone graft applications. The system is furthermore highly tunable by the employment and mixing of various comonomers, enabling the manufacturing of specialized materials. Comparing the described silyl ether-based materials to other materials aiming for the same application highlights its benefits. As mentioned in the introductory section, other cleavable motifs, such as boronic esters, had been considered for bone tissue engineering applications in the past. Boronic ester-based monomers used in thiol–ene photopolymers have been reported to yield stiff and hydrolytically degradable materials.^17^ Although degradability per se is crucial for the application as a bone scaffold, the described rapid degradation of the boronic ester-based material (more than 60% mass loss after 2 weeks) may be too fast to sufficiently support the forming bone tissue. The silyl ether-based photopolymer described herein exhibits a more moderate degradation speed with a mass loss of 8% after 2 weeks. Furthermore, another advantage of silyl ether-based monomers is their improved printability as they are in a liquid state at rt, while the discussed formulations containing boronic ester-based monomers (solid state at rt) required handling at temperatures >50 °C to prevent reprecipitation of the monomer.

To develop the material platform described herein toward clinical application, further *in vitro* and also *in vivo* characterization will be performed, e.g., to assess the response of living tissue to silyl ether-based photopolymers. Moreover, hydroxyapatite filling of those photopolymers will be investigated to enhance osteoinductivity and mechanical properties.

## Supplementary Material



## Notes

^1^H and ^13^C NMR spectra for TSE, NSE, TCH and NM-dimer. HMBC spectra (^1^H, ^29^Si) and (^1^H, ^13^C) for NSE and TSE compounds. ^13^C NMR spectra for TSE precursors NMTA and NMT. Experimental details for the synthesis of TCH and NM-dimer. UHPLC-MS for TSE, NSE, and NM-dimer. Further details and results for photoreactivity studies, tensile tests, degradation study, cytotoxicity, and additive manufacturing (PDF)
