# Supplementary material for: Cleavable Silyl Ether Monomers with Elevated Thermomechanical Properties for Bone Regeneration
Source: ACS Appl Bio Mater. 2025 Nov 7;8(11):9840–53. doi: 10.1021/acsabm.5c01174 (PMC12628333; doi:10.1021/acsabm.5c01174)
Supplement: Supplementary file 1 [file mt5c01174_si_001.pdf]

Supporting Information

Cleavable silyl ether monomers with elevated thermomechanical properties for bone regeneration

Tina Gurmann[1,2,3]*, Judith Krauß*[2]*, Theresa Ammann*[2]*, Thomas Koch*[4]*, Martin Frauenlob*[2]*,*

Robert Liska[2,3]*, Stefan Baudis*[1,2,3]*

AUTHOR ADDRESS:

[1]Christian Doppler Laboratory for Advanced Polymers for Biomaterials and 3D Printing, 1060 Vienna, Austria.

[2]Institute of Applied Synthetic Chemistry, Technische Universität Wien, 1060 Vienna, Austria.

[3]Austrian Cluster for Tissue Regeneration, 1200 Vienna, Austria.

[4]Institute of Materials Science and Technology, Technische Universität Wien, 1060 Vienna, Austria.

*stefan.baudis@tuwien.ac.at

Table of Contents

1. ^{13}C-NMR spectrum of NMTA 3
2. ^{13}C-NMR spectrum of NMT 4
3. ^{1}H-, ^{13}C-NMR spectra and (^{1}H, ^{13}C) and (^{1}H, ^{29}Si) HMBC spectra of TSE 5
4. ^{1}H-, ^{13}C-NMR spectra and (^{1}H, ^{13}C) and (^{1}H, ^{29}Si) HMBC spectra of NSE 8
5. Synthesis of TACH 11
6. Synthesis of TCH 12
7. Synthesis of degradation product NM-dimer 14
8. Purity of compounds 17
9. ATR-FTIR spectra 21
10. Tensile tests 23
11. Degradability of polymer networks 24
12. Solubility of monomers and cytocompatibility 27
13. Additive manufacturing 30
14. Literature 32

1. ^{13}C-NMR spectrum of NMTA

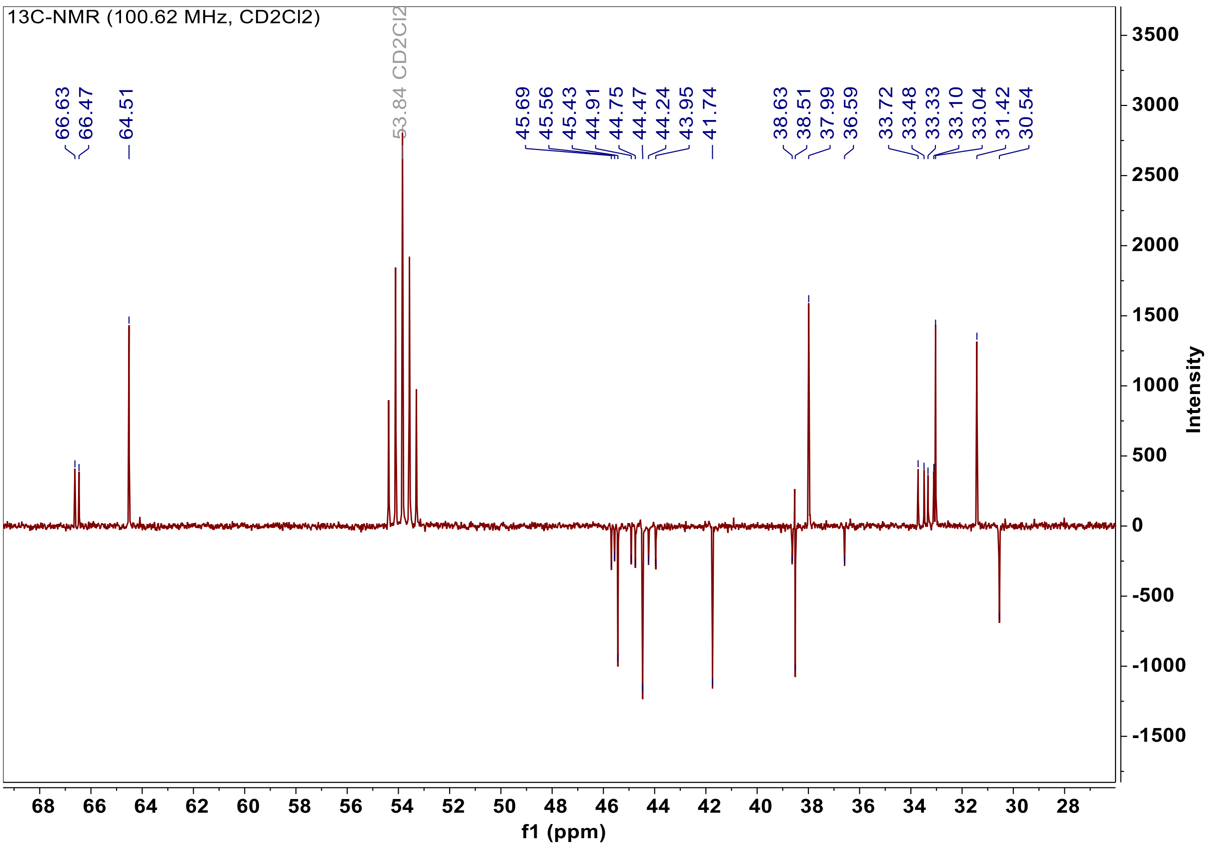

Figure S1: ^{13}C-NMR of NMTA. The educt (5-norbornene-2-yl)methanol was received as a mixture of endo/exo isomers, which led to splitting of the carbon signals.

2. ^{13}C-NMR spectrum of NMT

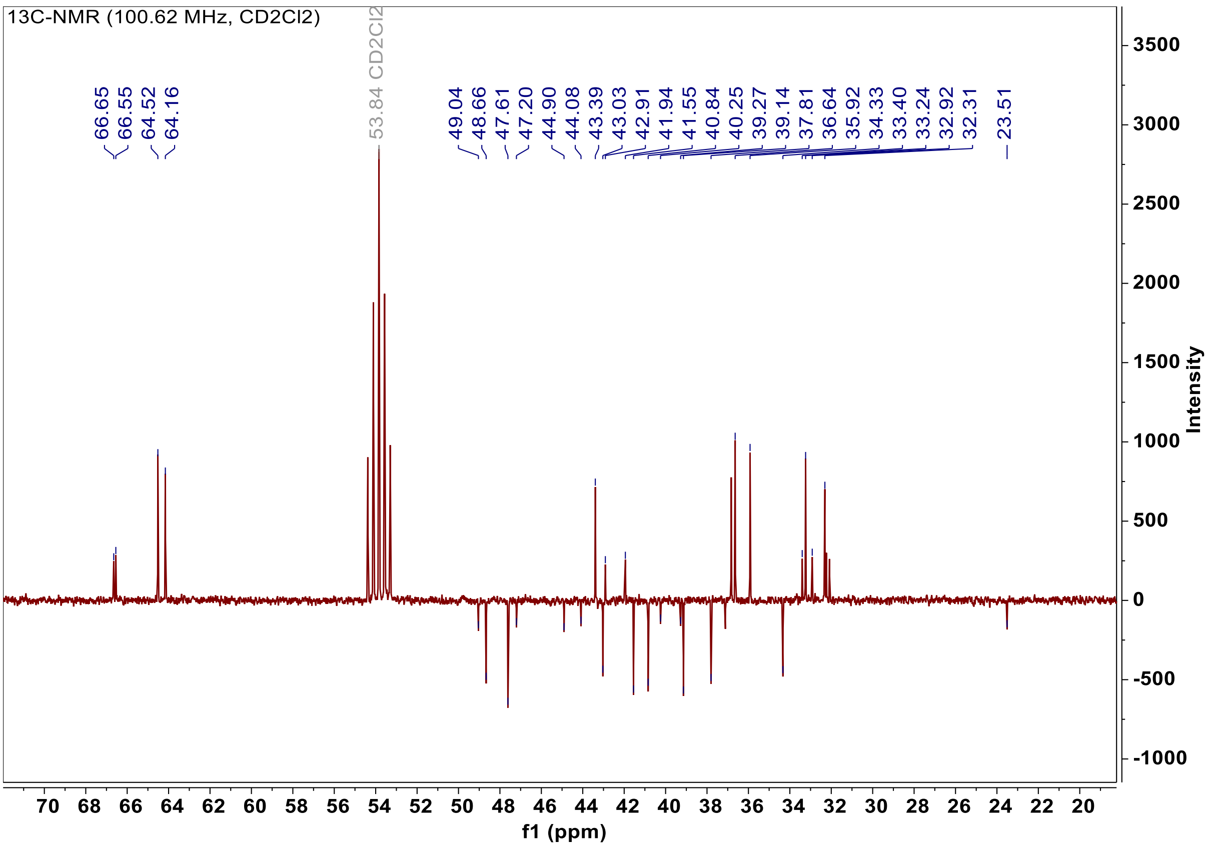

Figure S2: ^{13}C-NMR of NMT. The educt (5-norbornene-2-yl)methanol was received as a mixture of endo/exo isomers, which led to splitting of the carbon signals.

3. ¹H-, ¹³C-NMR spectra and (¹H, ¹³C) and (¹H, ²⁹Si) HMBC spectra of TSE

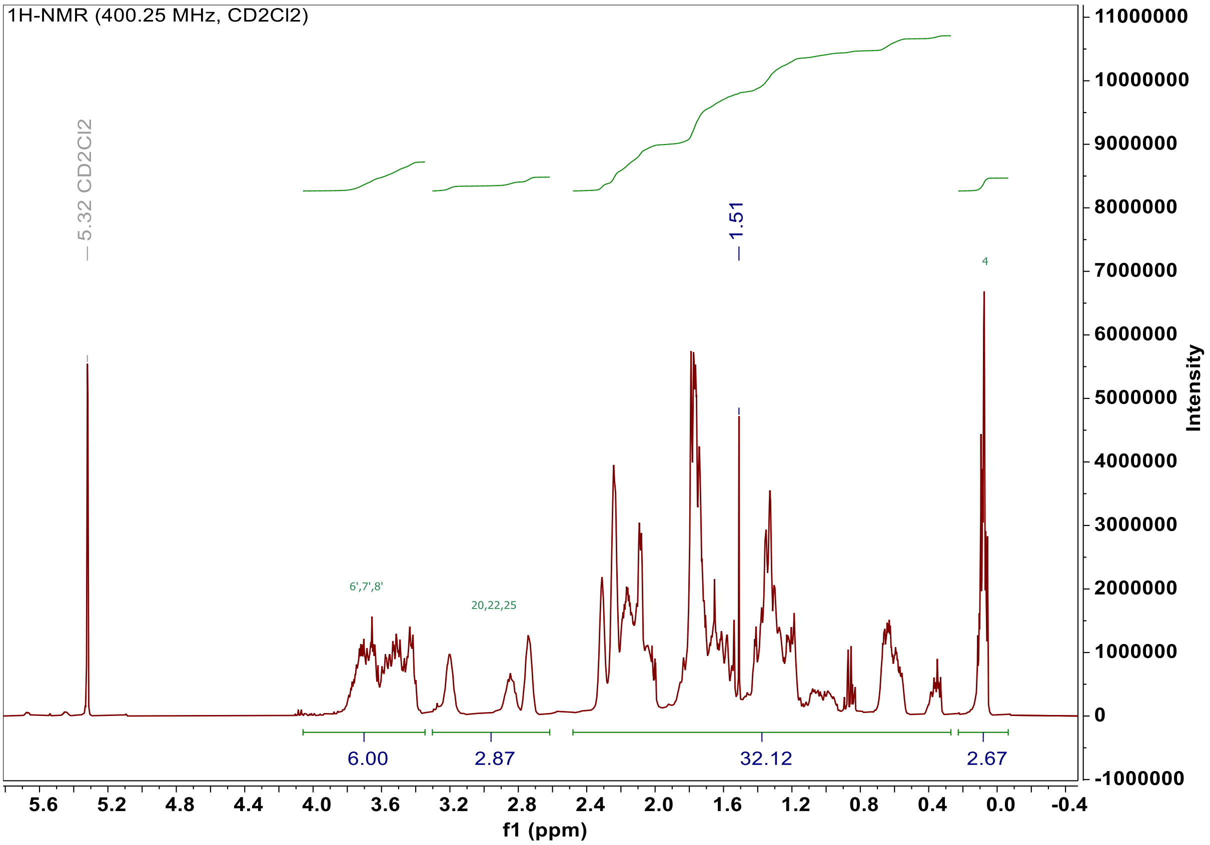

Figure S3: ¹H-NMR of TSE. The educt (5-norbornene-2-yl)methanol was received as a mixture of endo/exo isomers, which resulted in further coupling upon introduction of the thiol groups during NMT synthesis. Therefore, assignment of the peaks from δ2.39 – 0.28 ppm was not possible except for the thiol peak at δ1.51 ppm.

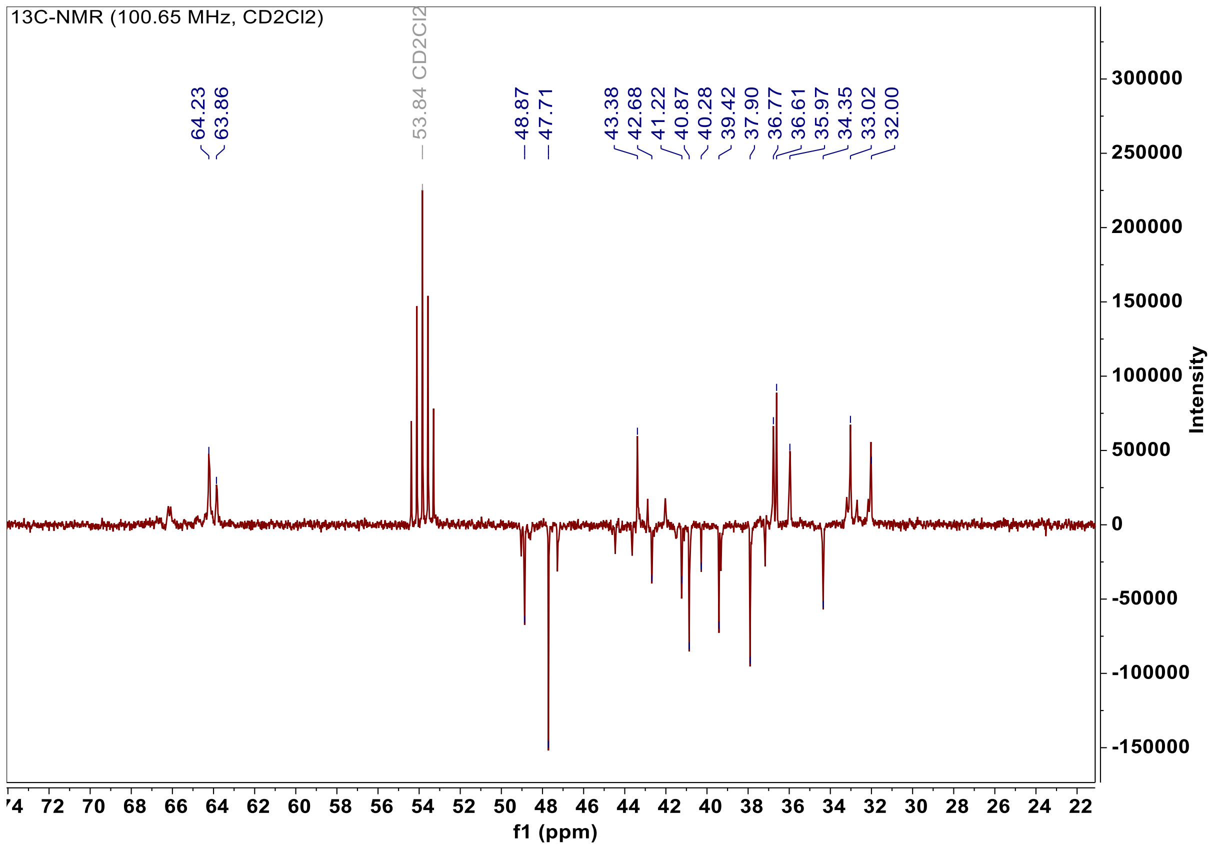

Figure S4: ^{13}C-NMR of TSE. The educt (5-norbornene-2-yl)methanol was received as a mixture of endo/exo isomers, which led to splitting of the carbon signals.

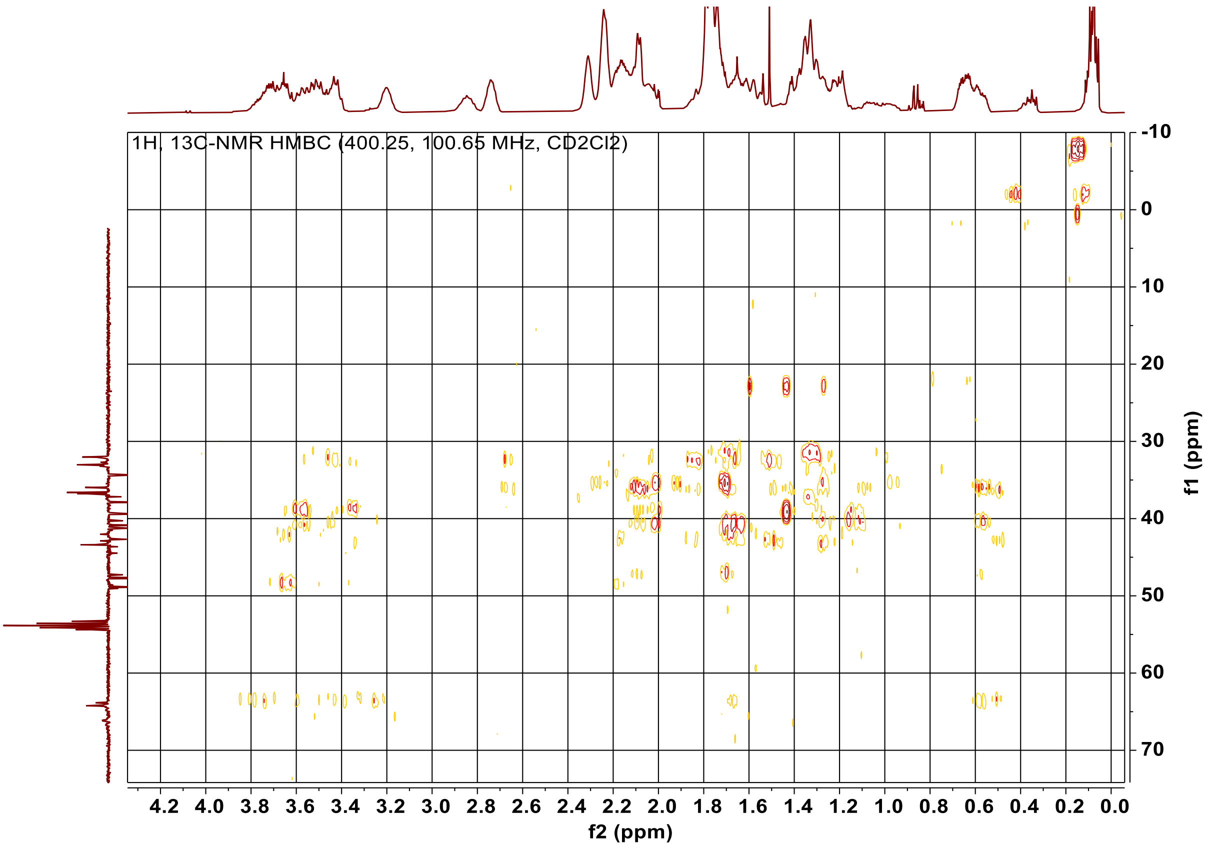

Figure S5: HMBC spectrum (^{1}H, ^{13}C) of TSE

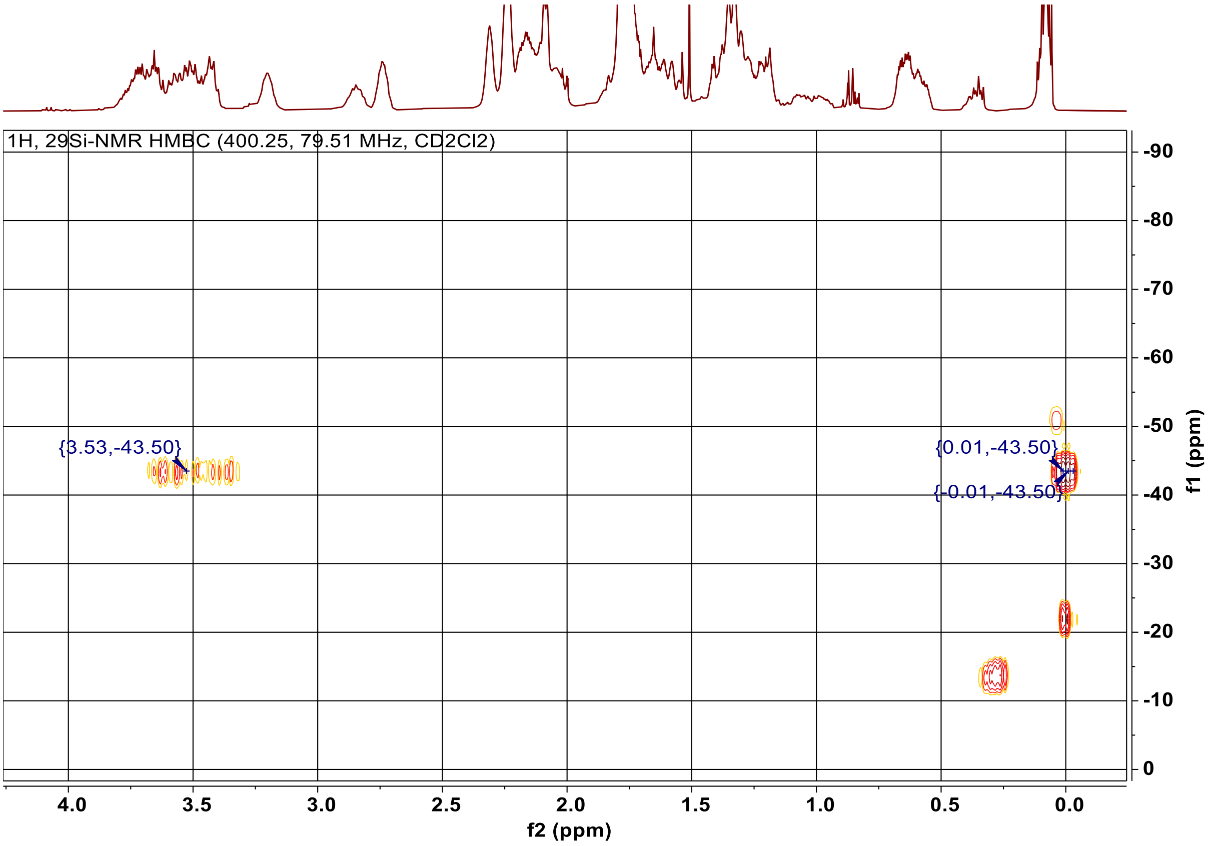

Figure S6: HMBC spectrum (^{1}H, ^{29}Si) of NSE. The crosspeak at {3.53, -43.5} proved converstion of NMT and the chlorosilane. {0.01, -43.5} stands for the crosscoupling of the -CH_3 group and the silicon atom. {-0.1, -21.99} stems most likely from a grease impurity, as shifts from δ-20 to δ-25 ppm indicate -O-SiRR'-O- siloxane species. {0.26, -13.78} stems most likely from a chain end of the grease impurity, as shifts from δ-10 to δ-16 ppm indicate -O-SiRR'-OH siloxane species.[1]

4. ^{1}H-, ^{13}C-NMR spectra and (^{1}H, ^{13}C) and (^{1}H, ^{29}Si) HMBC spectra of NSE

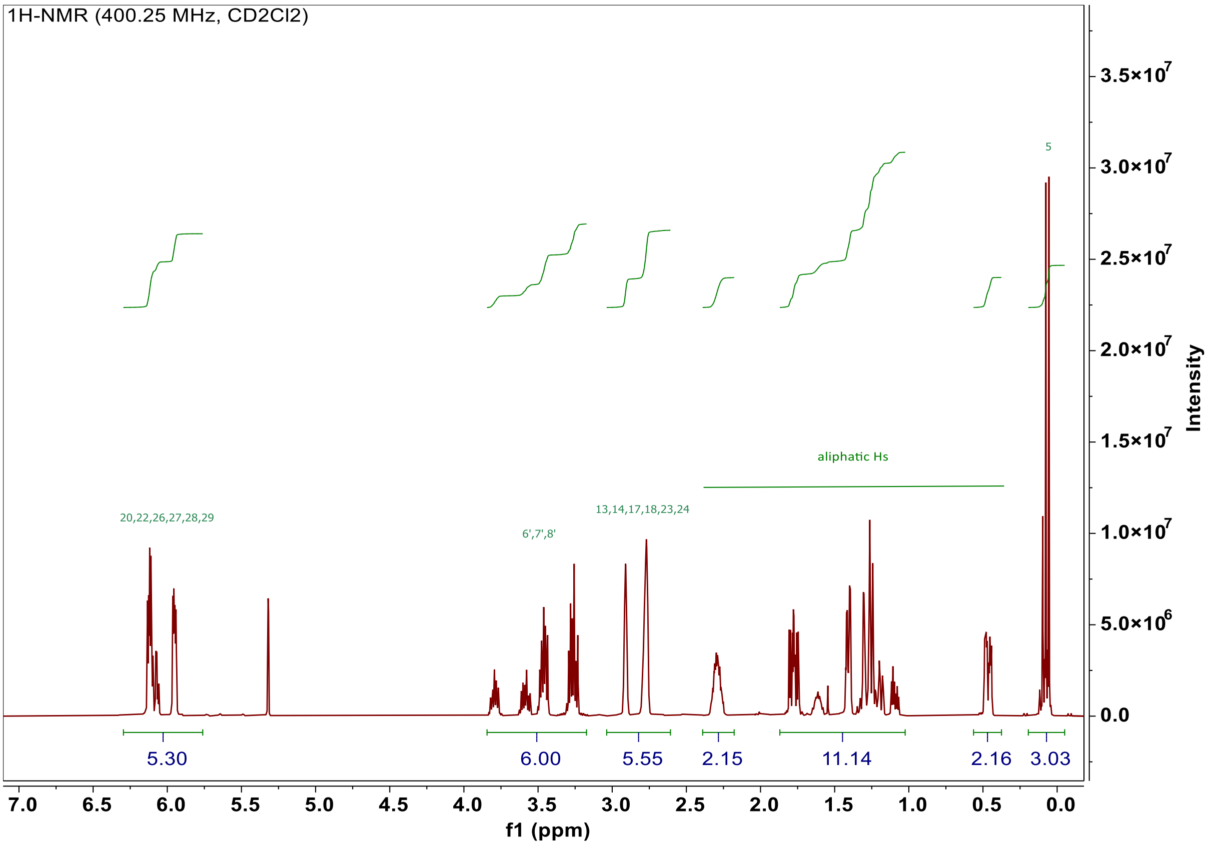

Figure S7: ^{1}H NMR of NSE. The educt (5-norbornene-2-yl)methanol was received as a mixture of endo/exo isomers, which resulted in further coupling. Therefore, assignment of the peaks from δ2.4 – 0.33 ppm was not possible.

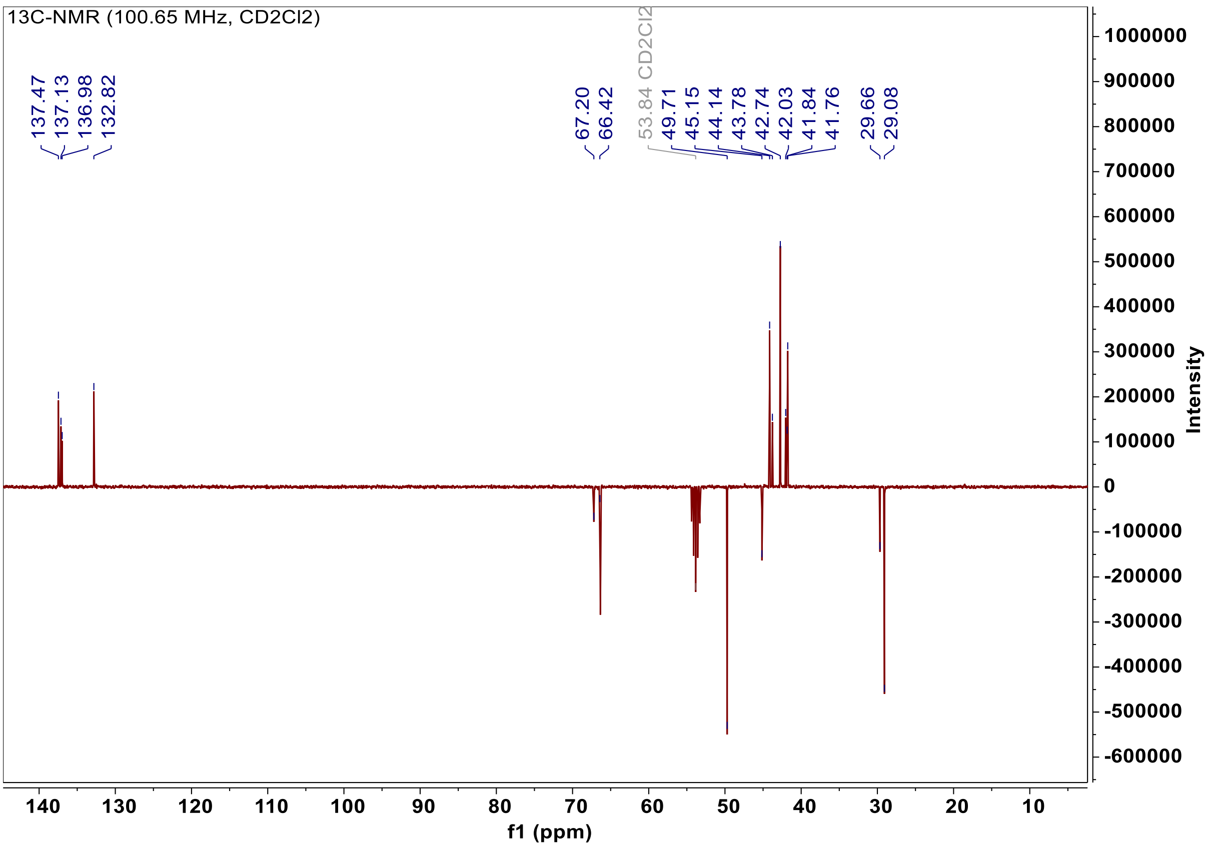

Figure S8: ^{13}C NMR of NSE. The educt (5-norbornene-2-yl)methanol was received as a mixture of endo/exo isomers, which led to splitting of the carbon signals.

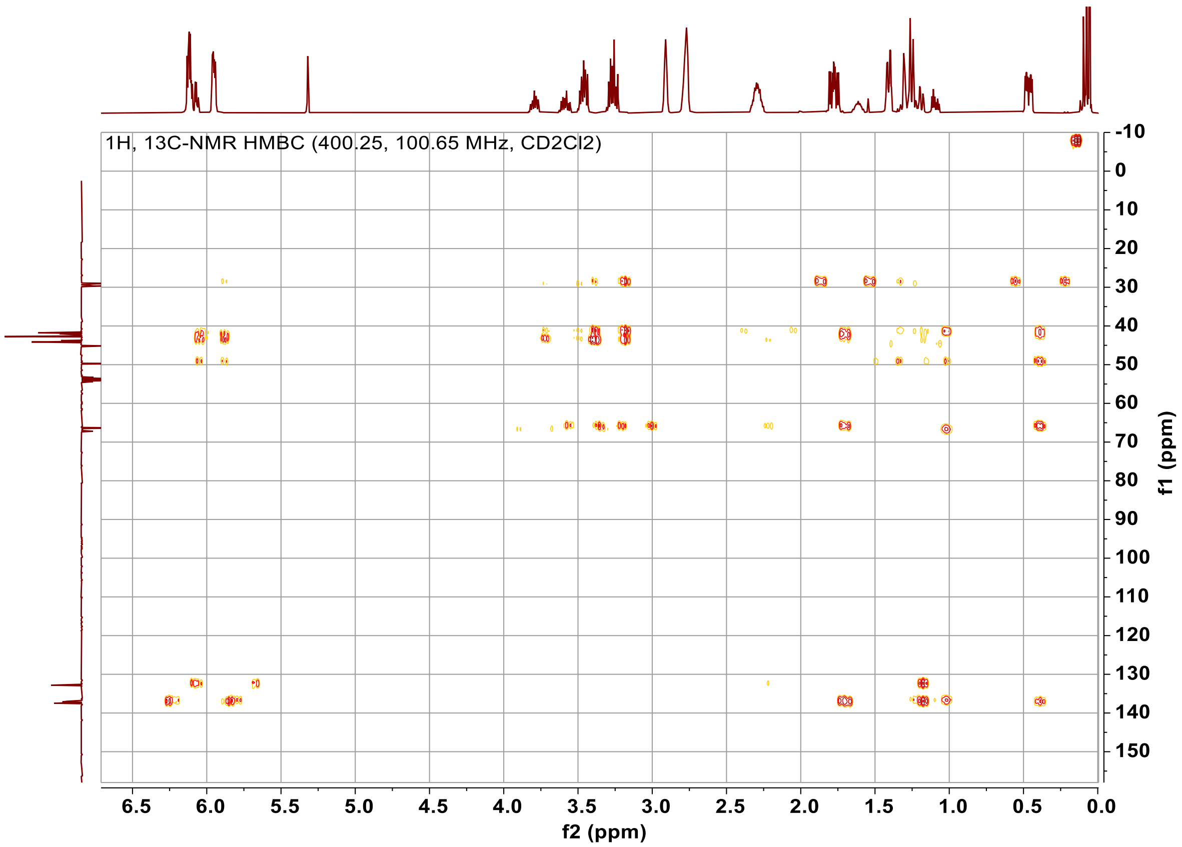

Figure S9: HMBC spectrum (^{1}H, ^{13}C) of NSE

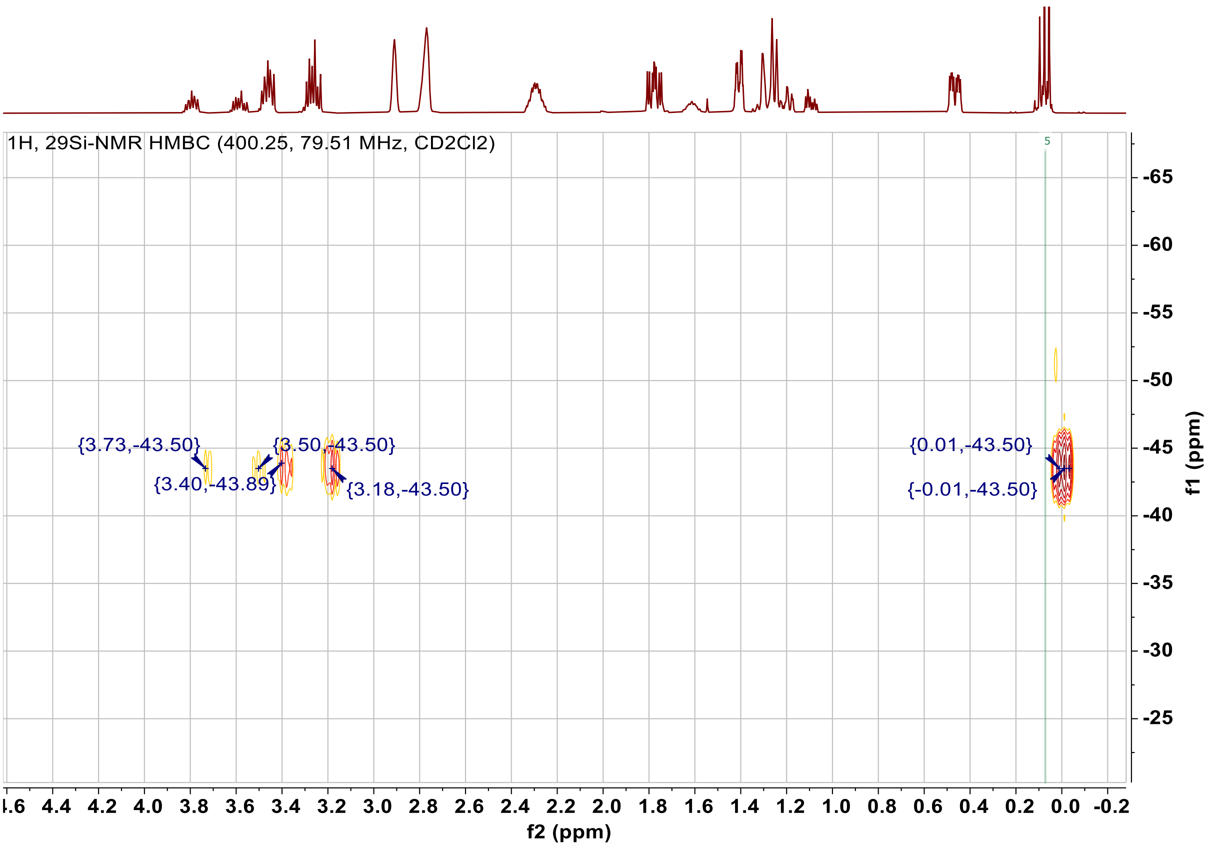

Figure S10: HMBC spectrum (^{1}H, ^{29}Si) of NSE. Crosspeaks at {3.73, -43.5}, {3.5, -43.5}, {3.4, -43.5} and {3.18, -43.5} prove converstion of (5-norbornene-2-yl)methanol and the chlorosilane. {0.01, -43.5} stands for the crosscoupling of the -CH_3 group and the silicon atom

5. Synthesis of TACH

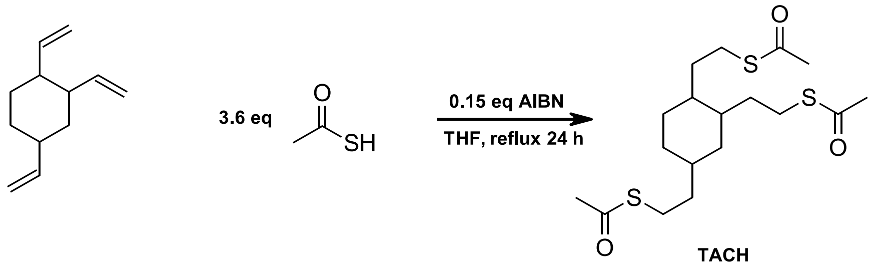

Scheme S1: Synthesis of TACH

The literature-known monomer precursor for TCH, TACH, was prepared in an analogous manner as previously described for NMTA and was therefore also adapted from literature.[2, 3] 1,2,4-Trivinylcyclohexane (30 g, 1 eq, 184.9 mmol) was dissolved in 190 mL dry THF

together with thioacetic acid (50.7 g, 3.6 eq, 665.6 mmol) and AIBN (4.6 g, 0.15 eq, 27.7 mmol) in a 500 mL three-necked flask. After purging the solution with argon for 30 minutes, the contents of the flask were heated to reflux for 24 h. After this, the flask was cooled to r.t. and 80 mL 1 M sodium carbonate solution was added. The mixture was extracted with DCM for three times (200/80/80 mL). The combined organic phase was extracted first with 60 mL 1 M sodium hydroxide followed by 60 mL brine. Afterwards, the organic phase was dried using sodium sulfate (anhydrous) and the solvent was removed in vacuo. The product was received in quantitative yield as dark yellow oil, which was used for the next synthesis step without further purification.

^{1}H NMR (400 MHz, $CDCl_3$) δ 3.05 – 2.59 (m, 6H, -C<u>H</u>$_2$-S-), 2.37 – 2.14 (m, 9H, -C<u>H</u>$_3$), 1.90 – 0.56 (m, 15H, aliphatic Hs).

6. Synthesis of TCH

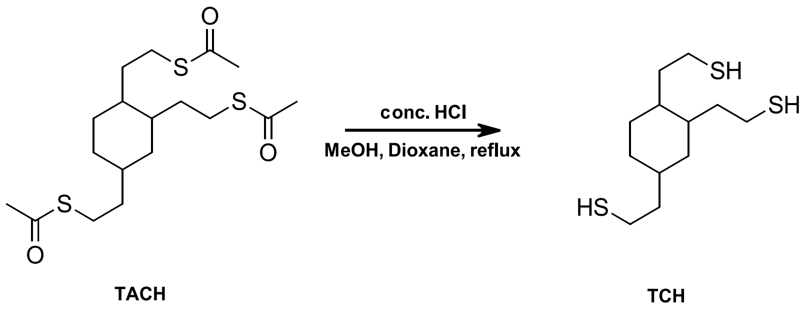

Scheme S2: Synthesis of TCH

Hydrolysis of TACH was performed as described for NMT.[2, 3] For this, TACH (74.0 g, 1 eq, 189.4 mmol) was transferred to a 1000 mL three-necked flask and diluted with 330 mL methanol and 110 mL dioxane. Conc. HCl (76 mL) was added subsequently. The reaction solution was purged with argon for 30 minutes to prevent oxidation of the resulting free thiols. After this, the solution was refluxed for 24 h. 130 mL deionized water was added to the cooled reaction mixture, which was then extracted four times with DCM (250/250/100/100 mL). The combined organic phases were then washed twice with each 100 mL saturated

sodium bicarbonate solution and then once with 100 mL brine. The organic phase was further dried with sodium sulfate (anhydrous). After removing the solvent in vacuo, the crude product was received as orange oil. For purification, Kugelrohr distillation (200 °C, 0.03 mbar) was performed. The final product was received as colorless oil (38.4 g, 77 % yield).

^{1}**H NMR** (400 MHz, $CDCl_3$) δ 2.75 – 2.31 (m, 6H, -C<u>H</u>$_2$-SH), 1.90 – 0.54 (m, 18H, aliphatic Hs, -S<u>H</u>).

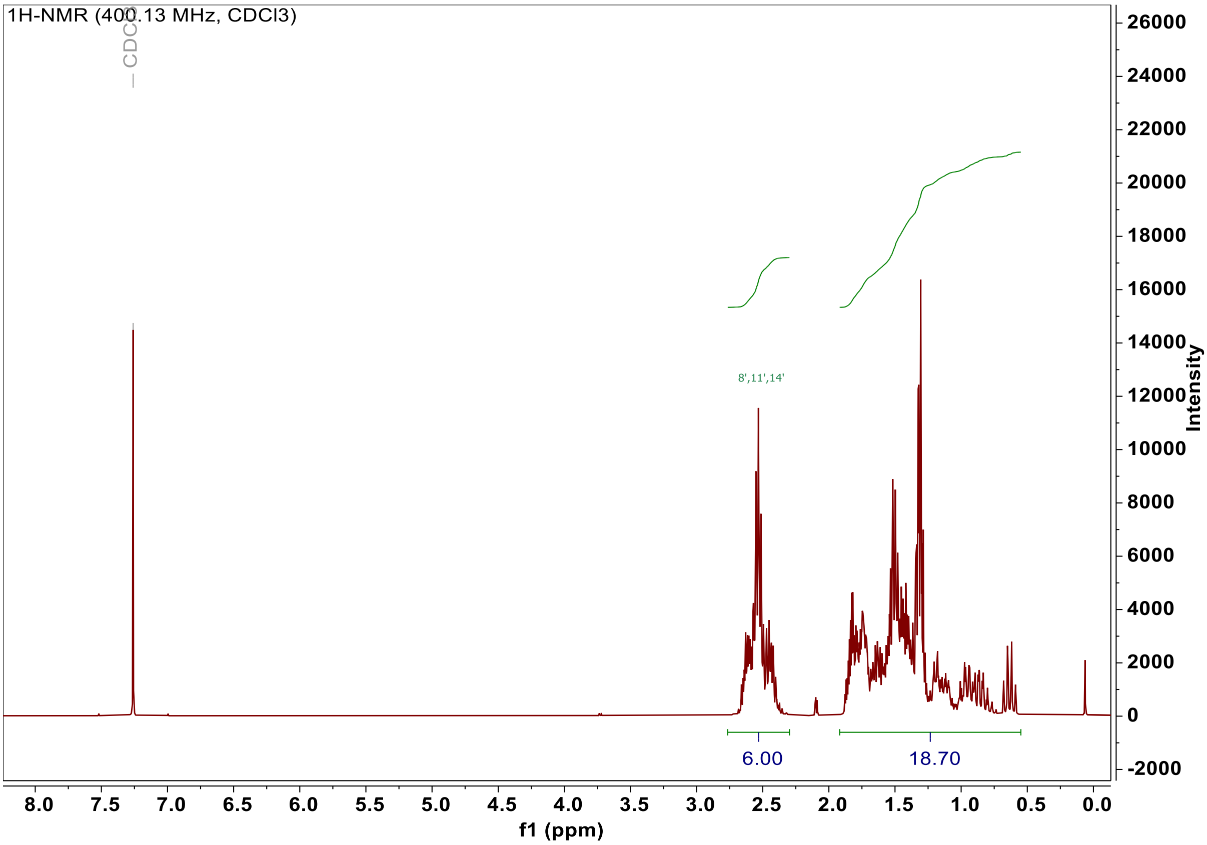

Figure S11: ^{1}H NMR of TCH

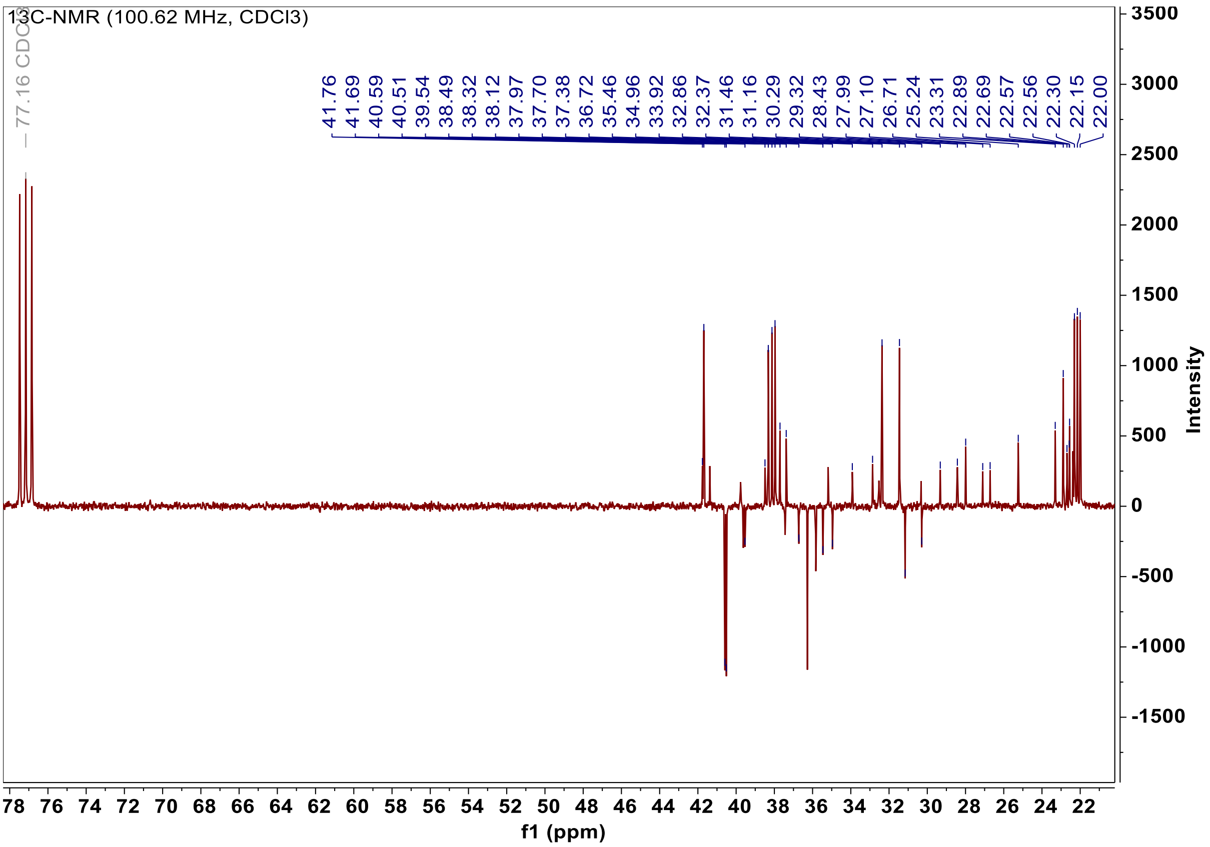

Figure S12: ^{13}C NMR (APT) of TCH

7. Synthesis of degradation product NM-dimer

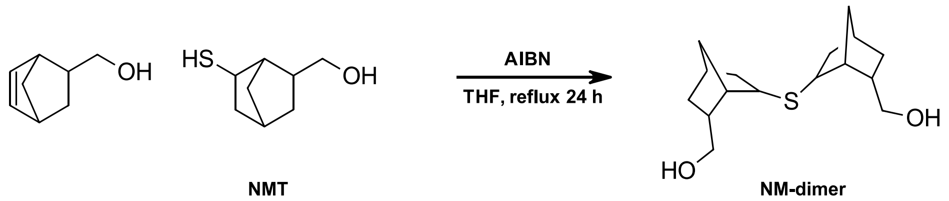

Scheme S3: Synthesis of degradation product NM-dimer

NM-dimer was synthesized starting with distilled (5-norbornene-2-yl)methanol (3 g, 1 eq, 24.2 mmol), NMT (3.8 g, 1 eq, 24.2 mmol) and AIBN (0.2 g, 0.05 eq, 1.2 mmol). Reactants were added to a three-neck round-bottom flask and suspended in 20 mL abs. THF. The reaction mixture was purged with argon for 30 min at room temperature and then heated to reflux. After 24 h, the reaction mixture cooled to r.t. and 20 mL 1 M Na_2CO_3 solution was added. The mixture was extracted three times with DCM (35/35/10 mL) and the combined organic phase

was washed with 10 mL aqueous 1 M NaOH and 10 mL brine. Then, the organic phase was dried over sodium sulfate (anhydrous), filtered and the solvent was removed under high vacuum to obtain the crude product as highly viscous, orange oil. Purification was performed using column chromatography with silica. Chromatography was started with 1:1 (PE:EE) until elution started for the first peak (impurity). Mobile phase was changed to 1:2 (PE:EE) until full elution. After that, pure ethyl acetate was used for elution of the second peak (product). Removal of the solvent on high vacuum at 85 °C gave the product as clear, solidified melt (3.6 g, 53 % yield).

R_f (silica, PE:EE = 1:1): 0.19

^{1}H NMR (400 MHz, CDCl3) δ 3.82 – 3.14 (m, 4H, -C<u>H</u>$_2$-OH), 3.10 – 0.38 (m, 22H, aliphatic Hs).

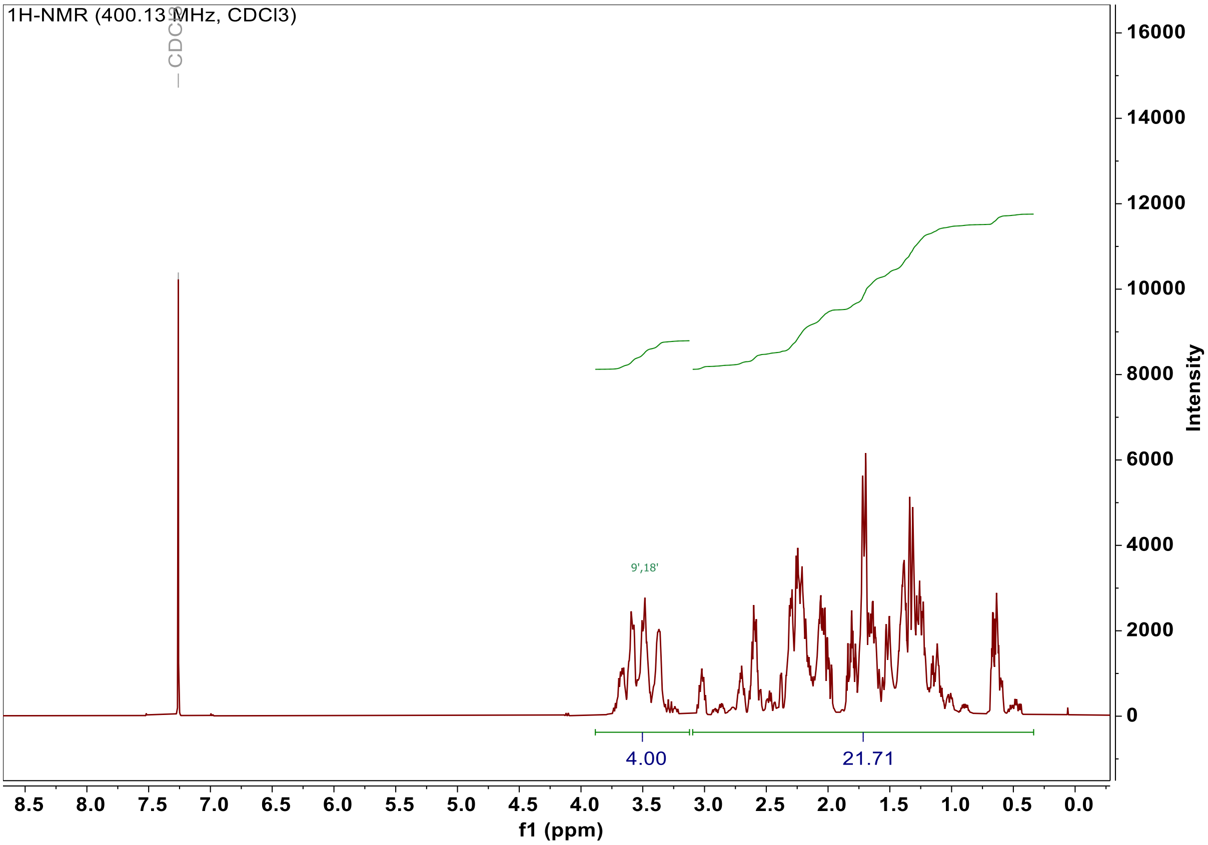

Figure S13: ^{1}H NMR of NM-dimer. The educt (5-norbornene-2-yl)methanol was received as a mixture of endo/exo isomers, which led to splitting and overlap of the signals. Therefore, further peak assignment was not possible.

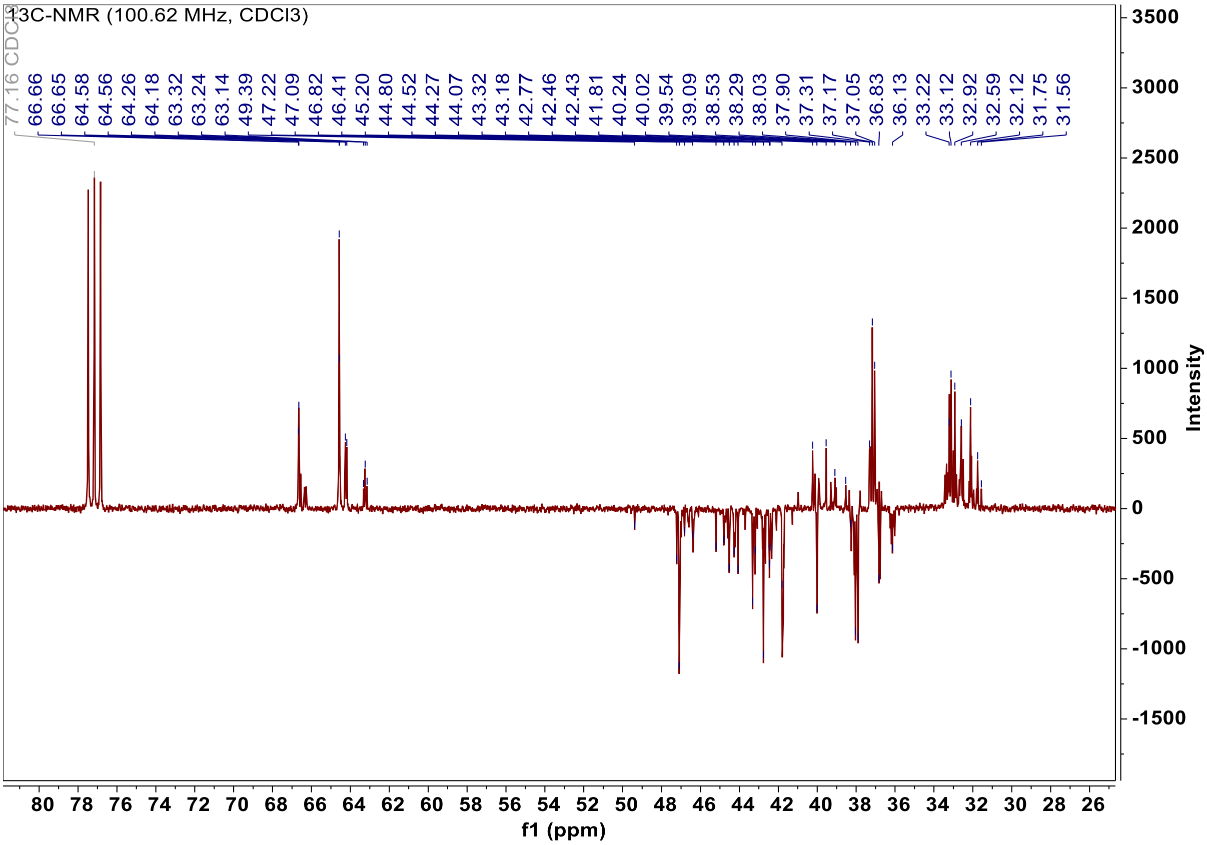

Figure S14: ^{13}C NMR of NM-dimer. The educt (5-norbornene-2-yl)methanol was received as a mixture of endo/exo isomers, which led to splitting of the carbon signals. With the introduction of the thiol for the NMT synthesis and further dimerization, a plethora of isomers was received, resulting in a high number of different carbon species.

8. Purity of compounds

UHPLC-MS measurements were performed on a Nexera 70 equipped with a Waters™ XBridge C8 Column (130Å, 3.5 µm, 3.0×50 mm) under basic conditions (for TSE and NSE) or under acidic conditions (NM-dimer) with ESI and APCI as MS ionization methods (LC-MS-2020) and ELS-2041 evaporative light scattering detector (JASCO®). Analytes were dissolved in 90 vol% HPLC-grade acetonitrile and 10 vol% HPLC-grade water (2 mg mL^{-1}). To avoid hydrolysis, measurements were performed within 30 minutes after sample preparation.

8.1.Purity of TSE

The chromatogram of TSE showed one distinctive peak, demonstrating the high purity of the tested substance. The corresponding mass spectrum showed the molecular ion peak at 513 m/z with high intensity. Furthermore, an adduct peak was also found at 559 m/z, which corresponds to a formic acid adduct ([M+46]).

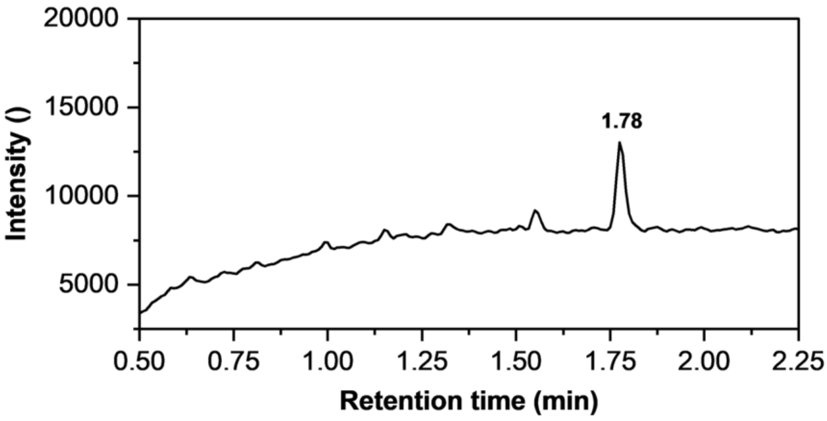

Figure S15: UHPLC-MS chromatogram (ELSD detection) of TSE

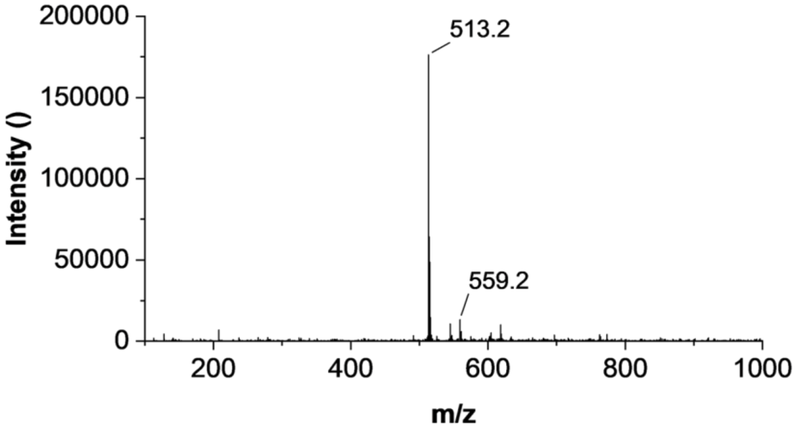

Figure S16: Mass spectrum (D-) at retention time 1.78 mins

8.2.Purity of NSE

NSE was also shown to be of high purity. The molecular ion peak was found here at 413 m/z in high intensity.

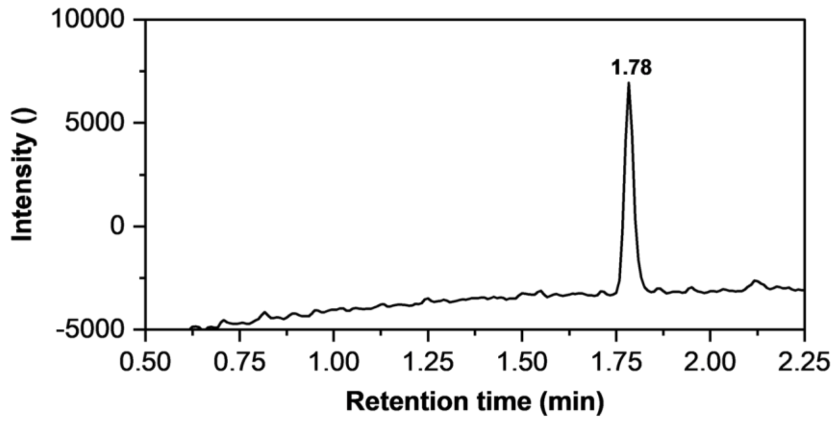

Figure S17: UHPLC-MS chromatogram (ELSD detection) of NSE

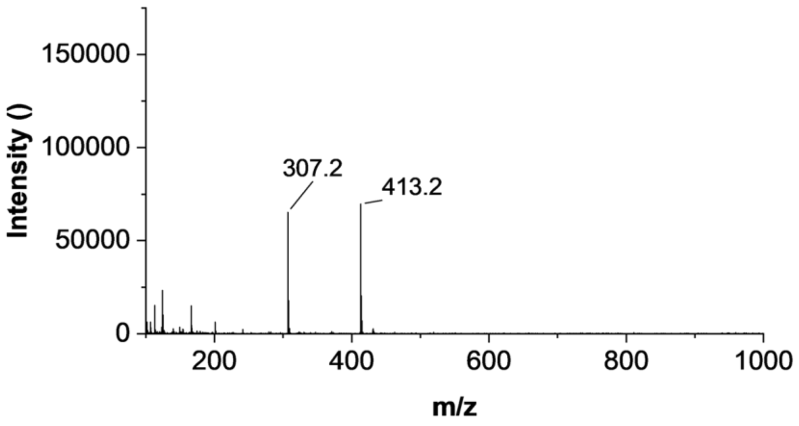

Figure S18: Mass spectrum (D+) at retention time 1.78 mins

8.3. Purity of NM-dimer

The chromatogram of NM-dimer (MW = 282.4 g/mol) showed three peaks that can be assigned to the different isomers that were obtained. The mass spectrum recorded during elution of the first peak showed the molecular ion peak as M+H at 283 m/z. Two adducts can be found that can be attributed to acetonitrile adducts, which is described in literature for alcohol and diols.[4] One at 324.1 m/z which corresponds to [M+ACN+H] and one at 365.2 m/z which corresponds to [M+2ACN+H]. The peak of [M+ACN+H] can also be found in the mass spectrum for t = 1.94 mins. With this, high purity of NM-dimer was shown.

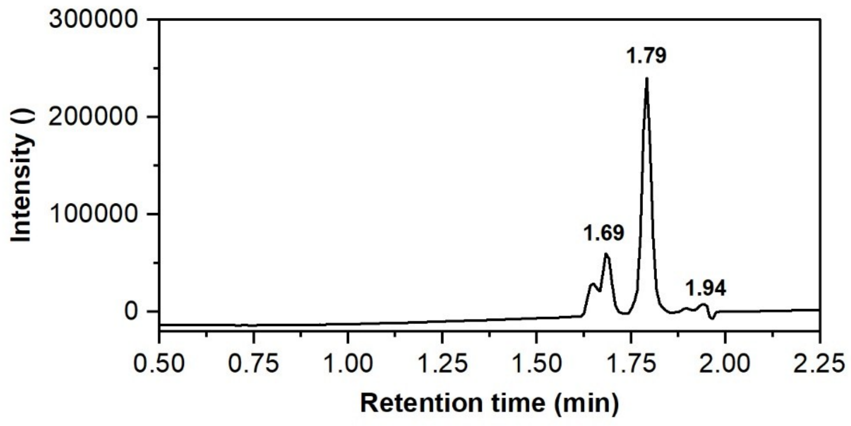

Figure S19: UHPLC-MS chromatogram (ELSD detection) of NM-dimer

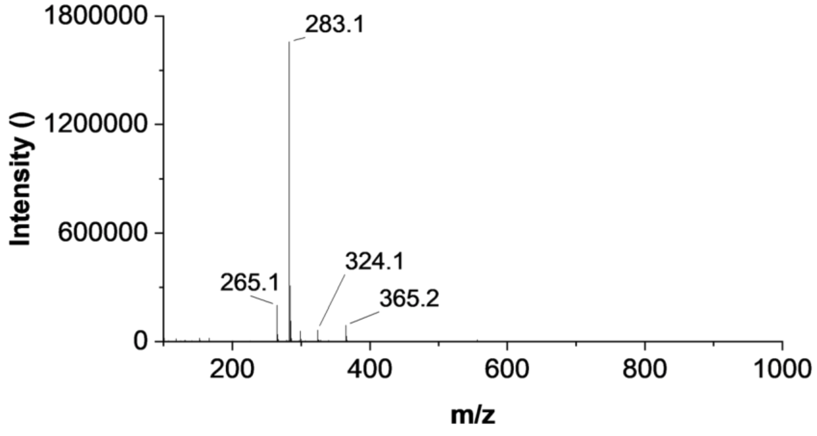

Figure S20: Mass spectrum (D+) at retention time 1.69 mins

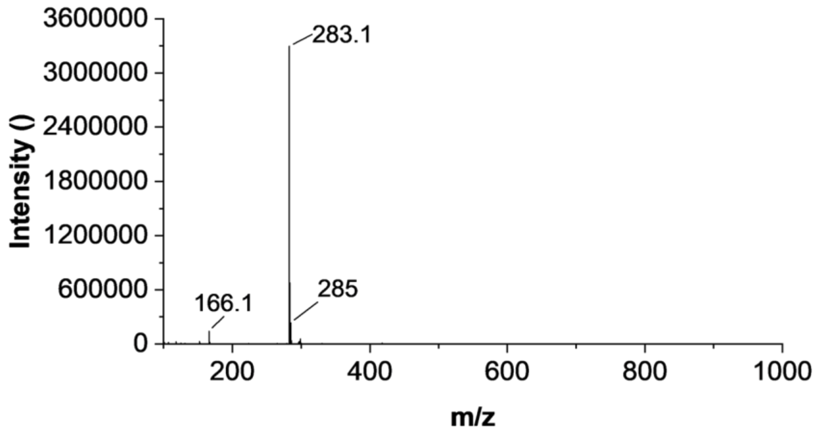

Figure S21: Mass spectrum (D+) at retention time 1.79 mins

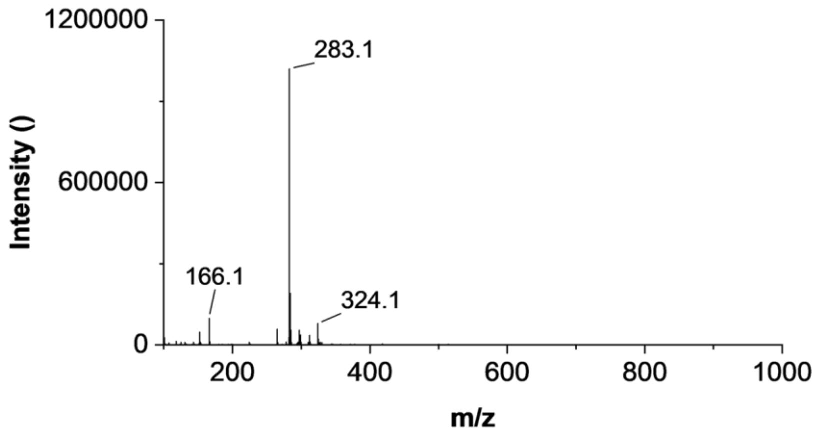

Figure S22: Mass spectrum (D+) at retention time 1.94 mins

9. ATR-FTIR spectra

For the calculation of double bond conversion, the CH stretch vibration (~ 3000 cm^{-1}, marked in purple) was used as a reference.

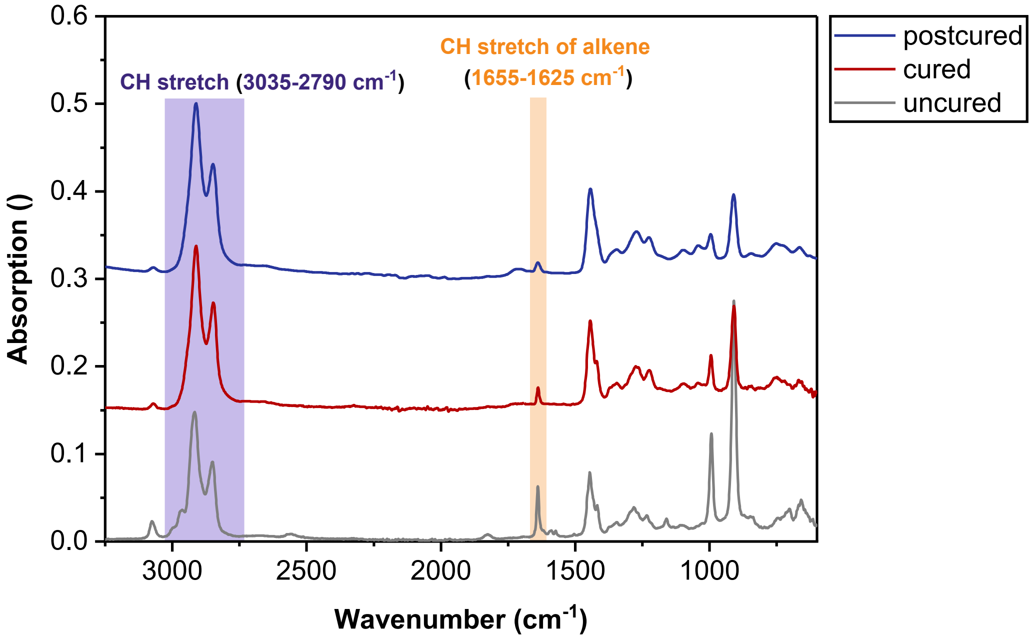

Figure S23: ATR-FTIR of VCH-TCH as uncured formulation, cured (photocured) polymer network and postcured polymer network. Curing procedures were carried out as described in experimental section.

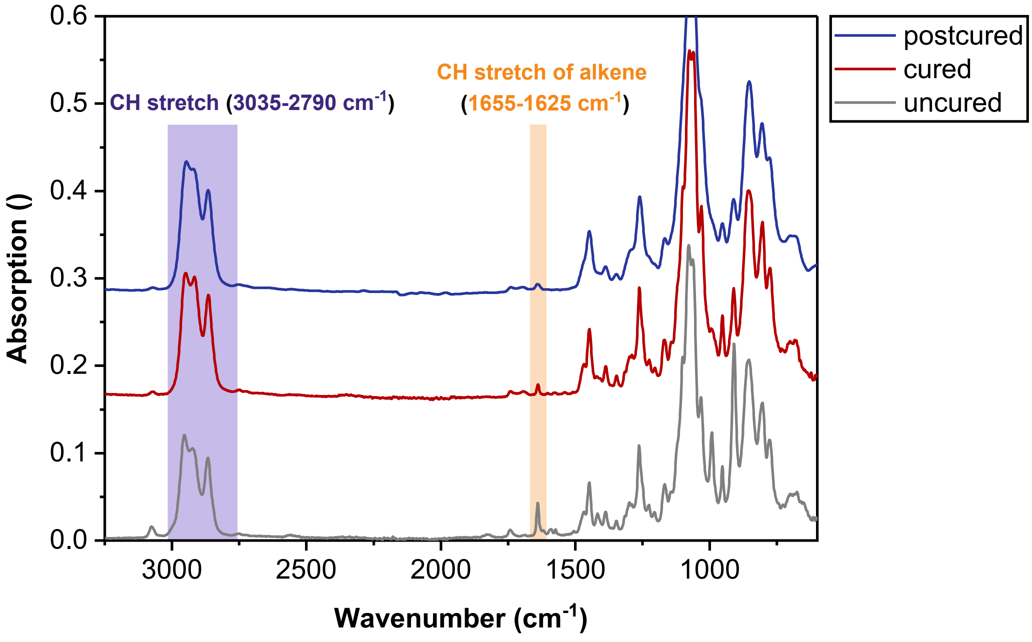

Figure S24: ATR-FTIR of VCH-TSE as uncured formulation, cured (photocured) polymer network and postcured polymer network. Curing procedures were carried out as described in experimental section.

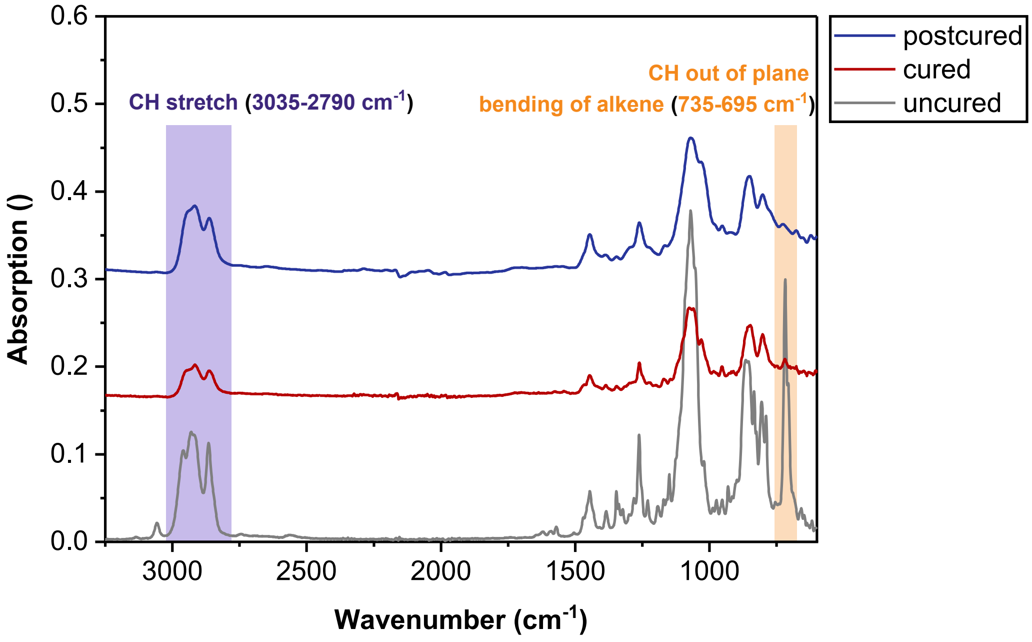

Figure S25: ATR-FTIR of NSE-TCH as uncured formulation, cured (photocured) polymer network and postcured polymer network. Curing procedures were carried out as described in experimental section.

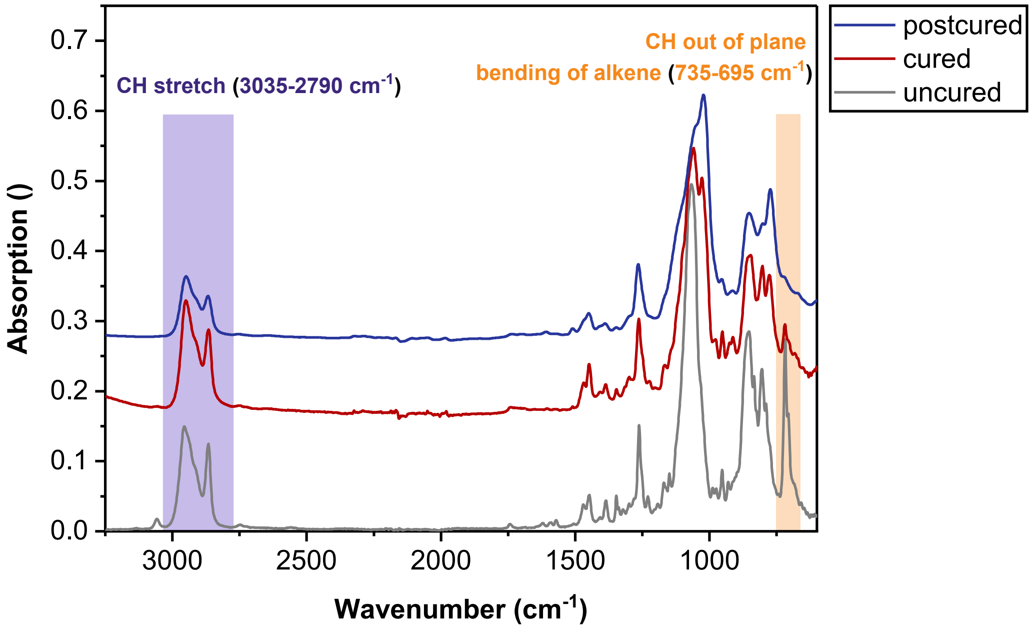

Figure S26: ATR-FTIR of NSE-TSE as uncured formulation, cured (photocured) polymer network and postcured polymer network. Curing procedures were carried out as described in experimental section.

10. Tensile tests

Table S1: Detailed results (mean ± standard deviation) of tensile tests

Polymer network	**σ_M (MPa)**	**ε_B (%)**	**U_T (MJ m^{-3})**
VCH-TCH	28 ± 1	39 ± 7	9.3 ± 1.4
NSE-TCH	53 ± 1	5 ± 1	1.7 ± 0.3
VCH-TSE	3 ± 0	327 ± 30	4.1 ± 0.6
NSE-TSE	35 ± 5	5 ± 1	0.9 ± 0.3

11.Degradability of polymer networks

Swelling was calculated according to the following equation:

$$Swelling\ (\%) = \frac{m_{swollen}(day)}{m_{dry}(day)} * 100$$

Table S2: Mean dry mass loss (%) and mean swelling (%) incl. respective standard deviations of cured polymer network VCH-TCH after storage at pH 4, 7.4 and 10 at 37 °C. Swelling (%) was calculated with respect to found dry masses at each timepoint.

	pH 4				pH 7.4				pH 10			
Time	Mean mass	STDEV mass	Mean swelling	STDEV swelling	Mean mass	STDEV mass	Mean swelling	STDEV swelling	Mean mass	STDEV mass	Mean swelling	STDEV swelling
(d)	(%)	(%)	(%)	(%)	(%)	(%)	(%)	(%)	(%)	(%)	(%)	(%)
0	100	0	100	0	100	0	100	0	100	0	100	0
2	97	1	103	1	98	1	102	0	99	1	102	1
7	96	1	105	1	98	1	103	1	97	0	104	0
14	97	0	104	0	98	1	102	1	97	1	104	0
30	96	0	105	1	97	1	104	1	98	1	103	1
61	96	0	105	0	97	1	104	1	96	1	105	1
90	96	0	104	0	97	1	104	2	96	1	105	1
180	96	0	105	1	96	1	105	2	96	1	105	1

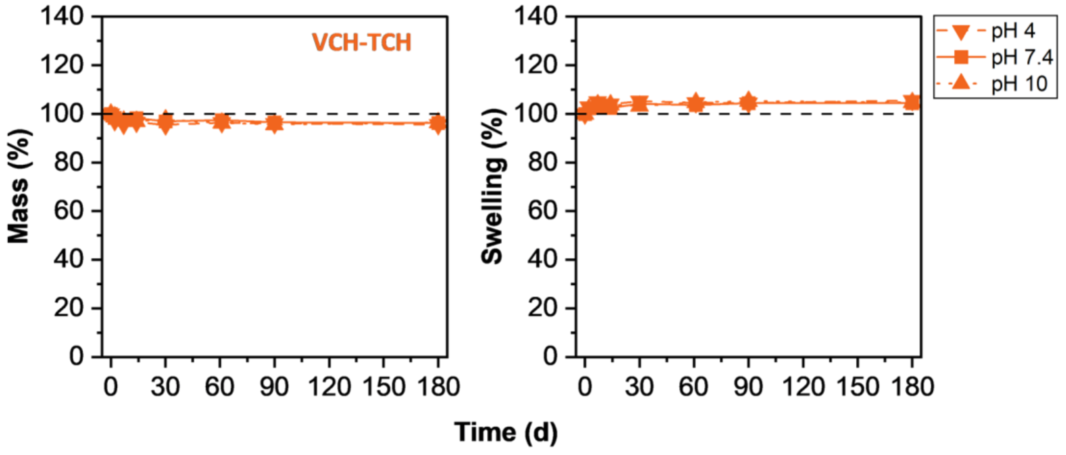

Figure S27: Dry mass loss (%) of cured polymer network VCH-TCH after storage at pH 4, 7.4 and 10 at 37 °C. Swelling (%) was calculated with respect to found dry masses at each timepoint.

Table S3: Mean dry mass loss (%) and mean swelling (%) incl. respective standard deviations of cured polymer network VCH-TSE after storage at pH 4, 7.4 and 10 at 37 °C. Swelling (%) was calculated with respect to found dry masses at each timepoint.

	pH 4				pH 7.4				pH 10			
Time	Mean mass	STDEV mass	Mean swelling	STDEV swelling	Mean mass	STDEV mass	Mean swelling	STDEV swelling	Mean mass	STDEV mass	Mean swelling	STDEV swelling
(d)	(%)	(%)	(%)	(%)	(%)	(%)	(%)	(%)	(%)	(%)	(%)	(%)
0	100	0	100	0	100	0	100	0	100	0	100	0
2	96	0	104	0	97	1	104	1	97	0	103	1
7	94	1	107	1	94	1	106	1	93	0	107	0
14	94	0	105	1	94	1	104	2	93	1	107	1
30	89	1	110	0	92	1	105	0	91	6	110	7
61	80	2	-	-	92	3	105	3	91	1	111	2
90	80	13	-	-	89	4	-	-	90	1	111	2
180	80	1	-	-	89	0	-	-	91	1	109	1

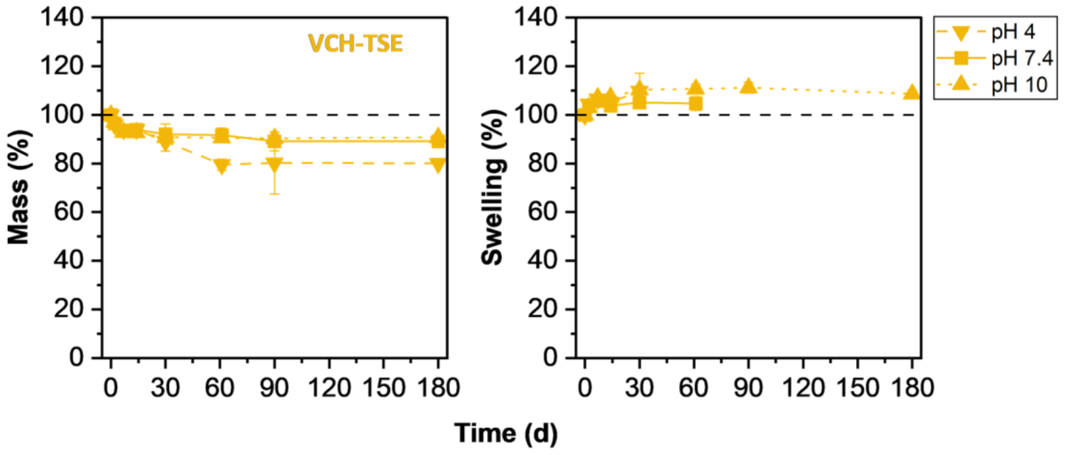

Figure S28: Dry mass loss (%) of cured polymer network VCH-TSE after storage at pH 4, 7.4 and 10 at 37 °C. Swelling (%) was calculated with respect to found dry masses at each timepoint.

Table S4: Mean dry mass loss (%) and mean swelling (%) incl. respective standard deviations of cured polymer network NSE-TCH after storage at pH 4, 7.4 and 10 at 37 °C. Swelling (%) was calculated with respect to found dry masses at each timepoint.

	pH 4				pH 7.4				pH 10			
Time	Mean mass	STDEV mass	Mean swelling	STDEV swelling	Mean mass	STDEV mass	Mean swelling	STDEV swelling	Mean mass	STDEV mass	Mean swelling	STDEV swelling

(d)	(%)	(%)	(%)	(%)	(%)	(%)	(%)	(%)	(%)	(%)	(%)	(%)
0	100	0	100	0	100	0	100	0	100	0	100	0
2	99	0	104	1	99	1	104	1	99	1	103	1
7	99	1	105	1	99	2	105	3	99	1	105	3
14	100	1	105	2	100	1	103	3	99	0	105	0
30	99	0	106	1	99	1	106	1	99	0	107	1
61	98	1	106	4	99	1	107	1	98	1	108	1
90	97	1	107	2	97	1	107	2	97	1	108	1
180	98	1	107	2	97	1	107	1	98	1	109	1

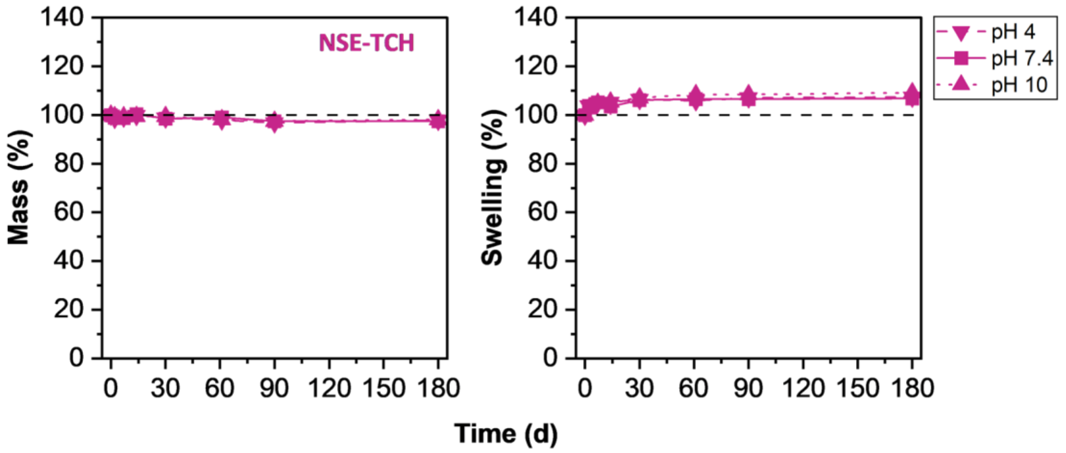

Figure S29: Dry mass loss (%) of cured polymer network NSE-TCH after storage at pH 4, 7.4 and 10 at 37 °C. Swelling (%) was calculated with respect to found dry masses at each timepoint.

Table S5: Mean dry mass loss (%) and mean swelling (%) incl. respective standard deviations of cured polymer network NSE-TSE after storage at pH 4, 7.4 and 10 at 37 °C. Swelling (%) was calculated with respect to found dry masses at each timepoint.

	pH 4				pH 7.4				pH 10			
Time	Mean mass	STDEV mass	Mean swelling	STDEV swelling	Mean mass	STDEV mass	Mean swelling	STDEV swelling	Mean mass	STDEV mass	Mean swelling	STDEV swelling
(d)	(%)	(%)	(%)	(%)	(%)	(%)	(%)	(%)	(%)	(%)	(%)	(%)
0	100	0	100	0	100	0	100	0	100	0	100	0
2	96	1	106	1	96	1	103	1	97	1	105	0
7	91	2	111	3	94	1	105	1	95	0	106	1
14	86	1	118	3	92	2	105	1	93	0	107	0
30	79	2	126	2	86	1	109	1	90	2	109	2
61	72	2	129	1	74	1	112	1	87	2	110	2
90	66	1	131	1	68	3	114	1	78	2	115	2

180	47	9	157	11	61	1	115	1	69	1	119	2

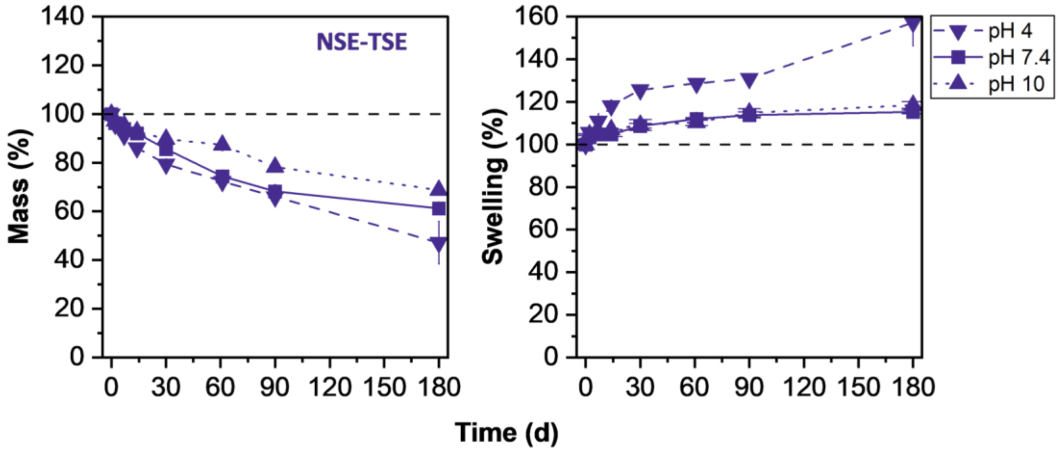

Figure S30: Dry mass loss (%) of cured polymer network NSE-TSE after storage at pH 4, 7.4 and 10 at 37 °C. Swelling (%) was calculated with respect to found dry masses at each timepoint.

12.Solubility of monomers and cytocompatibility

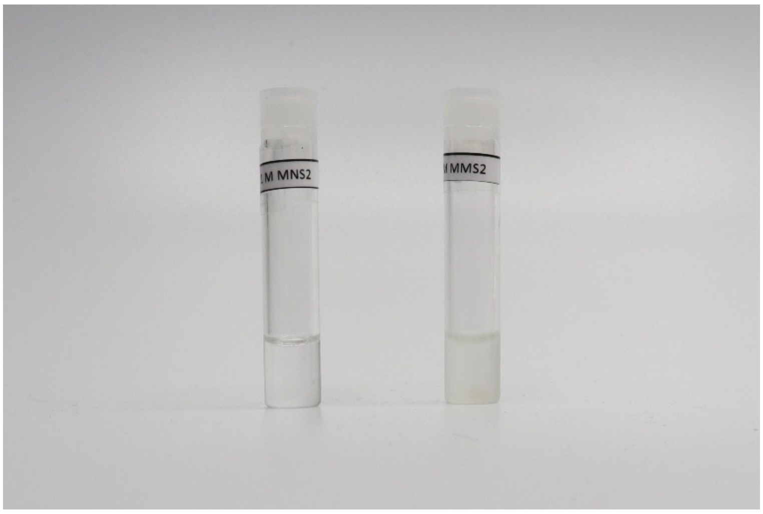

Figure S31: Solubility tests of NSE (left) and TSE (right) in the concentration of 1 M in DMSO at rt.

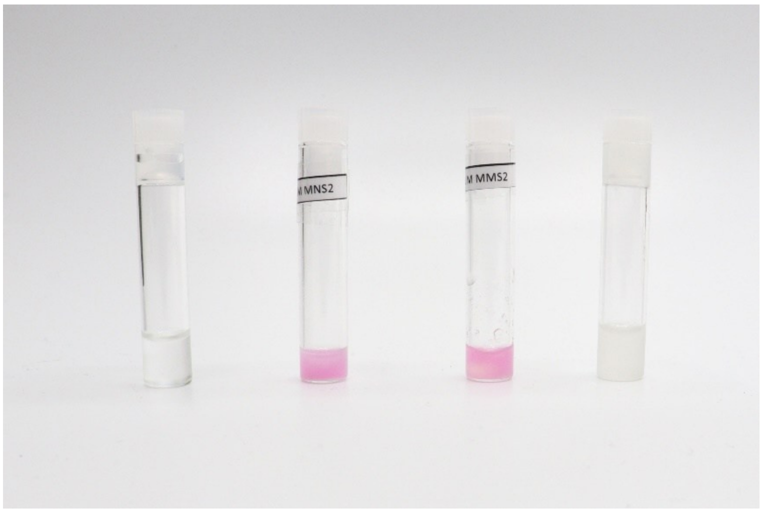

Figure S32: Solubility tests of NSE (two vials on left side) and TSE (two vials on right side) in the concentration of 1 M in DMSO (vials on the outer side) and diluted with DMEM to achieve final concentration of 0.1 mM after 24 h at 37 °C.

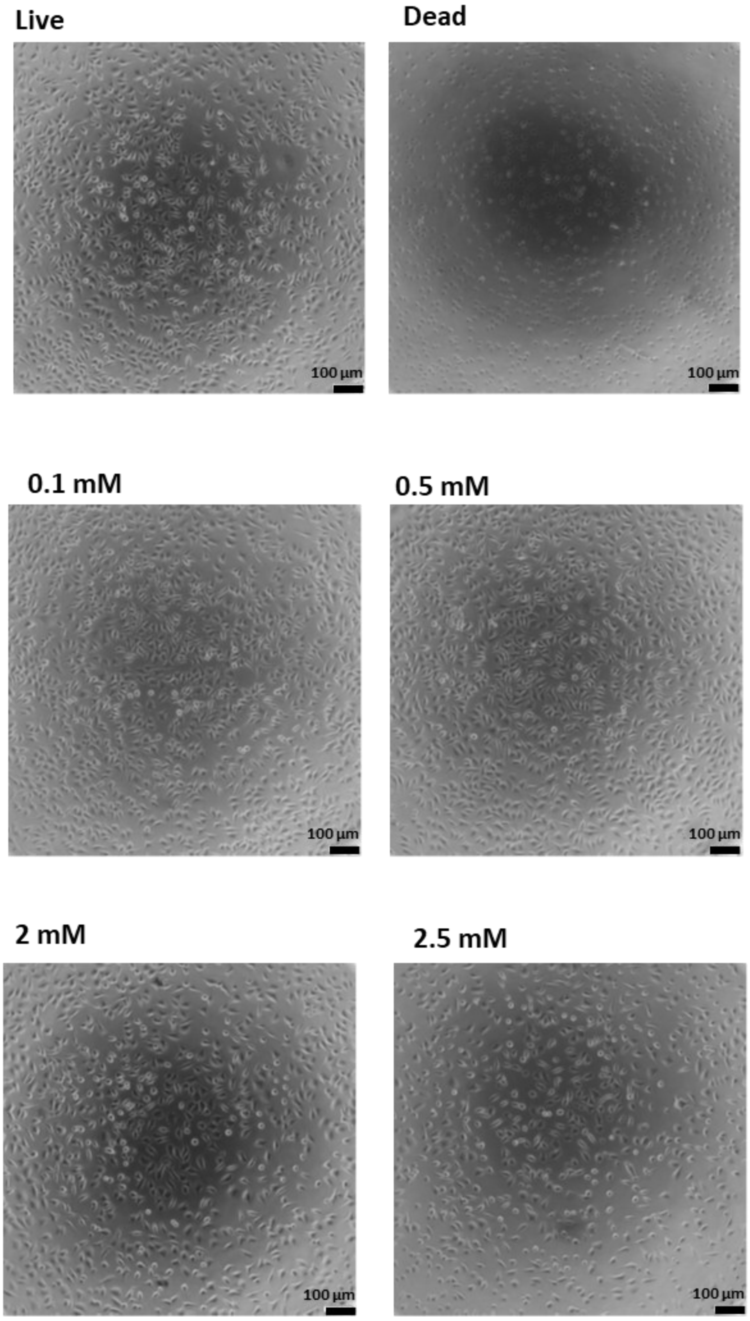

Figure S33: Microscopy images of cell cultures (NCTC clone 929 mouse fibroblast cells) after incubation with NM-dimer for 24 h

13.Additive manufacturing

Printing parameters were assessed by creating a three-point Jacobs' working curve.

Table S6: Tested parameters and curing depths C_d for generation of Jacobs' working curve

Light intensity (mW cm^{-2})	Irradiation time (s)	Energy (mJ cm^{-2})	C_d (µm)
**30**	3	90	44.3 ± 2.3
**40**		120	51.7 ± 10.7
**50**		150	58.7 ± 5.0

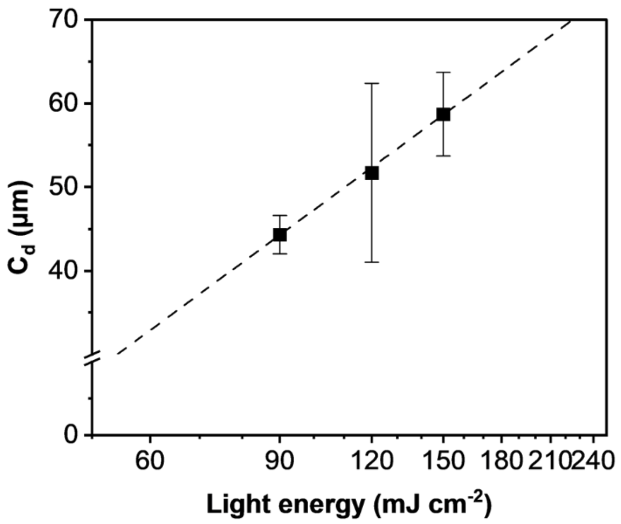

Figure S34: Jacobs' working curve

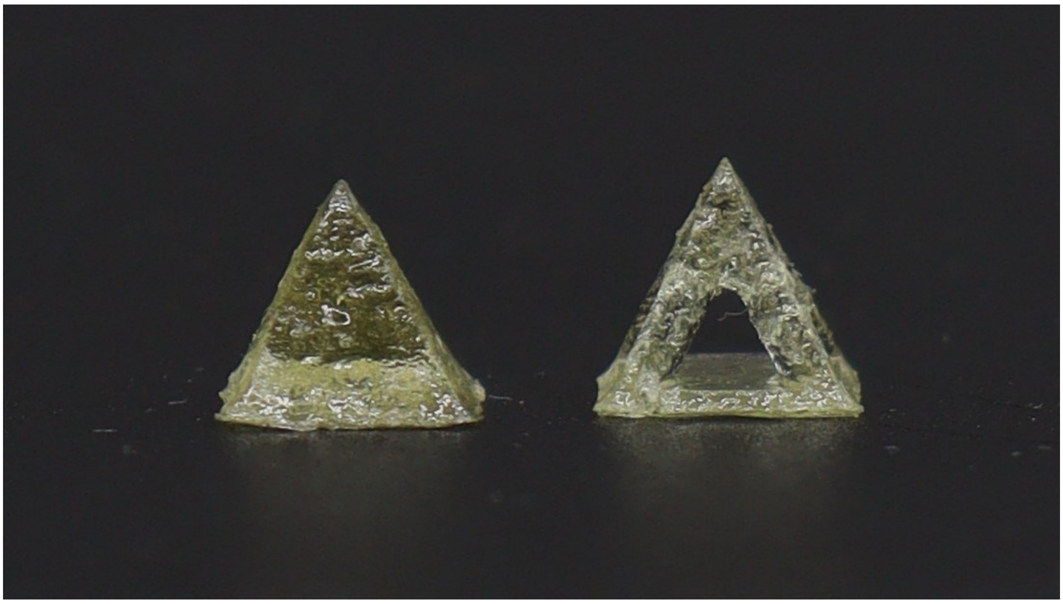

Figure S35: Full and hollow pyramids

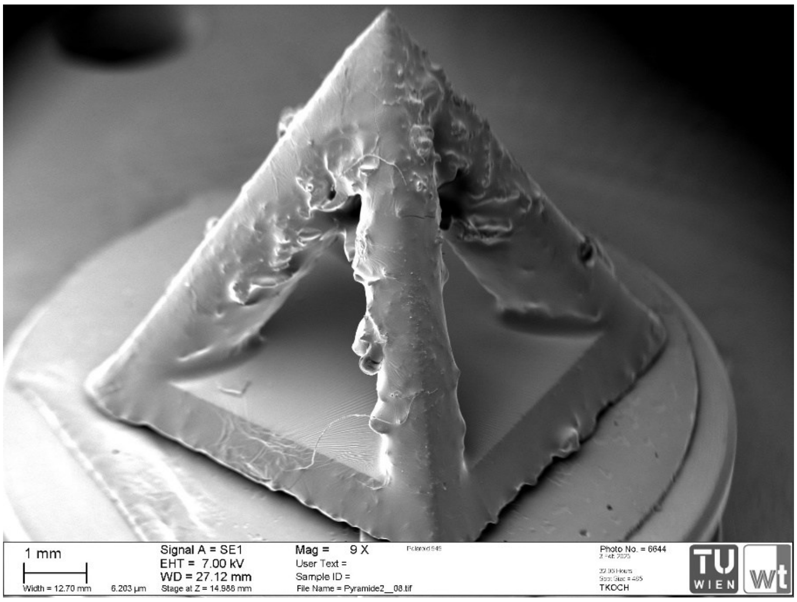

Figure S36: SEM image of hollow pyramid

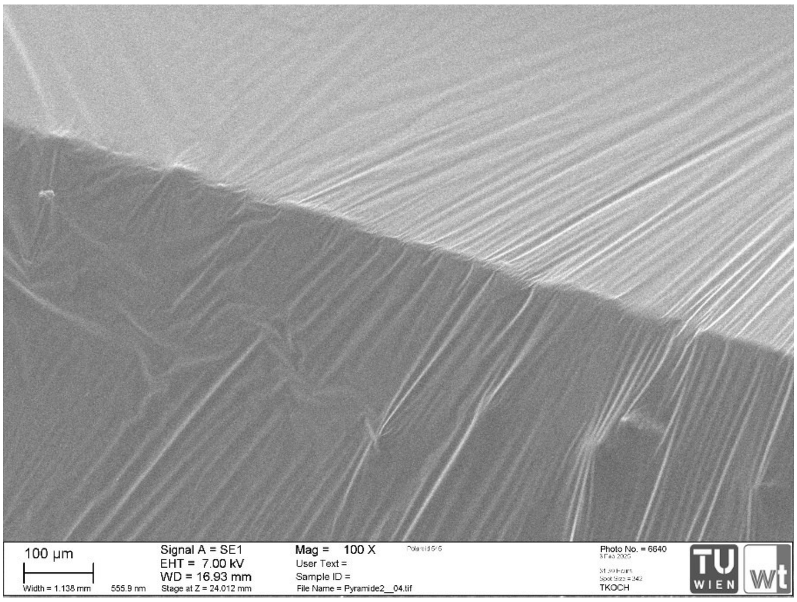

Figure S37: SEM image of hollow pyramid

14.Literature

1. Nam, K.-H.; Lee, T.-H.; Bae, B.-S.; Popall, M., Condensation reaction of 3-(methacryloxypropyl)-trimethoxysilane and diisobutylsilanediol in non-hydrolytic sol-gel process. *Journal of Sol-Gel Science and Technology* **2006,** *39* (3), 255-260.
2. Reinelt, S.; Tabatabai, M.; Moszner, N.; Fischer, U. K.; Utterodt, A.; Ritter, H., Synthesis and Photopolymerization of Thiol-Modified Triazine-Based Monomers and Oligomers for the Use in Thiol-Ene-Based Dental Composites. *Macromolecular Chemistry and Physics* **2014,** *215* (14), 1415-1425.
3. Van Damme, J.; van den Berg, O.; Brancart, J.; Vlaminck, L.; Huyck, C.; Van Assche, G.; Van Mele, B.; Du Prez, F., Anthracene-Based Thiol–Ene Networks with Thermo-Degradable and Photo-Reversible Properties. *Macromolecules* **2017,** *50* (5), 1930-1938.
4. Bogseth, R.; Edgcomb, E.; Jones, C. M.; Chess, E. K.; Hu, P., Acetonitrile adduct formation as a sensitive means for simple alcohol detection by LC-MS. *J Am Soc Mass Spectrom* **2014,** *25* (11), 1987-90.
